# Effects of 1-Year Intervention with a Mediterranean Diet on Plasma Fatty Acid Composition and Metabolic Syndrome in a Population at High Cardiovascular Risk

**DOI:** 10.1371/journal.pone.0085202

**Published:** 2014-03-20

**Authors:** Jordi Mayneris-Perxachs, Aleix Sala-Vila, Maribel Chisaguano, Ana I. Castellote, Ramón Estruch, María Isabel Covas, Montserrat Fitó, Jordi Salas-Salvadó, Miguel A. Martínez-González, Rosa Lamuela-Raventós, Emilio Ros, M. Carmen López-Sabater, for PREDIMED Study Investigators

**Affiliations:** 1 Department of Nutrition and Food Science-XARTA-INSA, Faculty of Pharmacy, University of Barcelona, Barcelona, Spain; 2 Lipid Clinic, Endocrinology and Nutrition Service, Biomedical Research Institute August Pi i Sunyer (IDIBAPS), Hospital Clínic, Barcelona, Spain; 3 Department of Internal Medicine, Hospital Clínic, Barcelona, Spain; 4 Cardiovascular Epidemiology Unit, Municipal Institut for Medical Research, Barcelona, Spain; 5 Human Nutrition Unit, Faculty of Medicine, IISPV, University Rovira i Virgili, Reus, Spain; 6 Department of Preventive Medicine and Public Health, School of Medicine, University of Navarra, Pamplona, Spain; 7 Physiopathology of Obesity and Nutrition (CIBEROBN), Madrid, Spain; Postgraduate Medical Institute & Hull York Medical School, University of Hull, United Kingdom

## Abstract

**Background & Aims:**

Metabolic syndrome (MetS) has become an important public concern due to its increasing prevalence. An altered fatty acid composition has been associated with MetS, but the Mediterranean diet has been shown to have a protective effect. The aim of the present study was to analyze the influence of a Mediterranean dietary pattern, as assessed by the biomarkers of food supplied, on the plasma fatty acid composition and its relation with MetS after 1 year of intervention.

**Methods:**

A total of 424 subjects were randomly selected from the PREDIMED randomized dietary trial after completing a 1-year intervention program. Participants aged 55 to 80 years and at high risk of cardiovascular disease were randomly assigned to three dietary interventions: Mediterranean diet supplemented with virgin olive oil or nuts, or a low-fat diet.

**Results:**

After 1 year of intervention participants in the virgin olive oil group showed significantly increased plasma concentrations of palmitic and oleic acids, but reduced proportions of margaric, stearic, and linoleic acids. In turn, subjects in the nut group showed significantly increased levels of palmitic, linoleic, and α-linolenic acids, but reduced proportions of myristic, margaric, palmitoleic, and dihommo-γ-linoleic acids. Increases in the biomarkers of foods supplied to the Mediterranean diet groups, i.e., oleic and α-linolenic acids, were beneficially associated with the incidence, reversion and prevalence of MetS. No weight changes were observed among participants.

**Conclusions:**

The nut and olive oil diets induced a fatty acid composition that has been shown to be beneficial in the face of MetS. Therefore, a Mediterranean diet rich in fats of vegetable origin may be a useful tool for the management of MetS without the need for concerns over weight gain due to its high fat content.

**Trial Registration:**

Controlled-Trials.com ISRCTN35739639

## Introduction

Metabolic syndrome (MetS) is defined as a clustering of interrelated metabolic risk factors that include dyslipidemia, hypertension, elevated fasting glucose and abdominal obesity [1]. The condition is widespread among adults from developed countries, with a prevalence of about 20 to 30%, or even higher, and with further increases in prevalence predicted due to the increasing incidence of obesity, diabetes and sedentary lifestyles [2], [3]. As people with MetS are at increased risk of developing both cardiovascular disease (CVD) [4] and type 2 diabetes [5], it has become an important public health concern and several organizations have attempted to formulate simple criteria for its diagnosis [3].

According to current guidelines, the first step in the management of MetS should emphasize therapeutic lifestyle modifications [6]. Although there is general agreement about reducing weight and increasing physical activity, a uniform consensus is lacking as to which diet is optimal [7]. However, epidemiological evidence suggests that individuals with MetS should adhere to a diet low in saturated and *trans* fats, cholesterol, sodium, and simple sugars, with an increased intake of fruits, vegetables, fish, and whole grains [6], [7]. Interestingly, these features resemble the principles of the traditional Mediterranean diet (MD) [8].

During the last few decades the MD has gained in popularity worldwide due to its reported contribution to lower rates of morbidity (particularly CVD, certain types of cancer, and other chronic conditions) and mortality, and the better health and quality of life of those who adhere to it [8]. Only a few cross-sectional, cohort and intervention studies have analyzed the relationship between MD adherence and the prevalence or incidence of MetS. However, the results from these studies have provided some evidence of the beneficial role of the MD in MetS and its components [9].

Although the dietary guidelines suggested for people presenting with MetS are similar to those of the MD and other healthy dietary patterns, the MD has a peculiarity of its own: a high content of total fat. This is the most distinguishing feature of the MD, with virgin olive oil (VOO) being the primary source of fat (providing 70–80% of the total fat). Unlike other oils however, the health-giving properties of VOO are derived not solely from its MUFA content (70–80% due to oleic acid), but also from minor components with great biological activity, including squalene, sterols, tocopherol, and highly bioavailable phenolic compounds [10]. Due to this double set of benefits, VOO favors a better lipid profile, reduces blood pressure (BP) levels, endothelial dysfunction, and the inflammatory and prothrombotic environment [11].

Tree nuts that have a unique fatty acid (FA) profile characterized by a high (MUFA+PUFA)/SFA ratio are also typical of the MD pattern. Although only some epidemiological studies have reported the possible benefits of nut consumption for type 2 diabetes and some cancers, their results have been remarkably consistent regarding CVD [12]. Apart from their favorable lipid profile, nuts contain vegetable proteins, L-arginine, fiber, folic acid, magnesium, copper, and different types of antioxidants such as flavonoids, polyphenols and tocopherols, which contribute to the cardioprotective properties of nuts via several mechanisms [13].

Compliance with a diet is often assessed using dietary surveys, which are associated with substantial measurement errors, whereas biomarkers of intake are potentially independent of these errors [14]. Therefore, an objective and accurate way to monitor fat quality is to record the plasma FA composition [14]. Individual plasma FA are generally expressed as a percentage of total FA. Therefore, changes in one FA may affect the levels of several other FA. Hence, considering the overall pattern of plasma FA may be a more useful measure of dietary quality than individual FA.

There is increasing evidence that the plasma FA composition may be influenced by diet [15]–[18] which may be useful for primary prevention, since an altered FA composition has been associated in several cross-sectional and observational studies with insulin resistance and diabetes [18], [19], obesity [20], CVD [21], and MetS [22]. However, studies assessing the effect that changes in the diet have on the FA profile and their relation with MetS remain scarce. Therefore, within the framework of a randomized controlled trial designed to compare the effects of two MD, one supplemented with VOO and another supplemented with nuts, with those of a low-fat diet on the cardiovascular outcomes among subjects at high risk of CVD (PREDIMED Study) [23], [24], the aim of this substudy was to analyze the effect of these diets on the plasma FA profile and its relation with MetS status after 1 year of intervention in participants recruited in the Barcelona north, Reus, and Pamplona centers.

## Methods

### Study design

The PREDIMED (PREvención con DIeta MEDiterránea) study is a large, parallel-group, multicenter, randomized, controlled, 5-year clinical trial aimed at assessing the effects of the Mediterranean diet on the primary prevention of cardiovascular disease. The main outcome is an aggregate of cardiovascular events (cardiovascular death, nonfatal myocardial infarction, or nonfatal stroke). The trial was registered with Current Controlled Trials, London (Identifier: ISRCTN35739639). The detailed protocol of the study has been published elsewhere [23], [24]. The protocol for this trial and supporting CONSORT checklist are available as supporting information; see Checklist S1 and Protocol S1, S2 and S3. The trial is currently ongoing with 7447 participants at high risk of CVD assigned randomly to three intervention groups: Mediterranean diet supplemented with virgin olive oil (MD+VOO), Mediterranean diet supplemented with mixed nuts (MD+nuts), or low-fat diet (Control). The present substudy was designed to assess the effects of the dietary interventions on the plasma FA profile and MetS status, two of the secondary outcomes of the PREDIMED study. We report, the results after 1 year's intervention in a subset of participants recruited in three centers of the PREDIMED trial (Barcelona North, Reus, and Pamplona).

### Ethics statement

The Institutional Review Board of the ten participating recruitment centers of the PREDIMED trial approved the study protocol and the participants signed an informed consent. In particular, the current substudy was approved by the Comité Etic d'Investigació Clínica de l'Hospital de Sant Joan, Reus (Tarragona), the Comisión de Etica de la Facultad de Medicina de la Universidad de Navarra, Pamplona (Navarra) and the Comité d'Etica e Investigació de l'Institut Municipal d'Investigació Mèdica (IMIM), Barcelona (Barcelona).

### Participants

**Figure 1:**
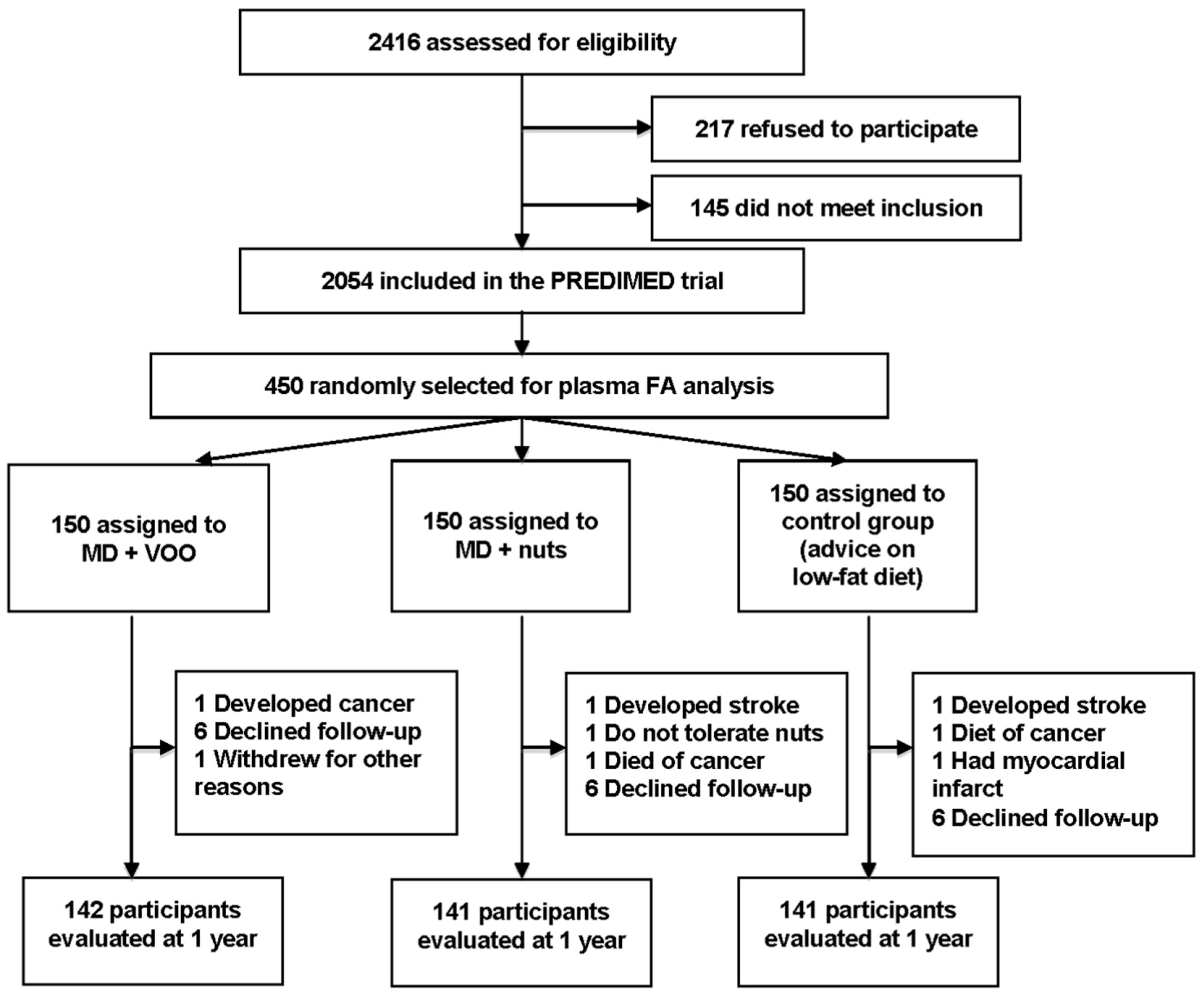
Trial flow-chart. Flow of patients through the present study involving three recruitment centres of the PREDIMED trial (Barcelona North, Reus, and Pamplona). FA indicates fatty acid; MD, Mediterranean diet; VOO, virgin olive oil.

Eligible participants for the PREDIMED Study were community-dwelling men, aged 55 to 80 years, and women, aged 60 to 80 years, who met at least one of the two following criteria: diagnosis of type 2 diabetes or presence of three or more CVD risk factors (smoking, hypertension, dyslipidemia, overweight or obesity, and family history of premature coronary heart disease (CHD)) [24]. For the present study, 2054 participants were recruited between June 2003 and May 2006 in the Barcelona (n = 320), Reus (n = 678) and Navarra (n = 1056) centers, three of the ten recruitment nodes of the PREDIMED trial. After a screening visit, participants were randomly assigned to one of three diet groups by means of a computer-generated random-number sequence. The plasma FA composition was measured in 22% of the participants who entered this substudy at baseline (n = 450). These participants were selected at random, and matched by age and sex. However, as some participants were lost during follow-up (Figure 1), the final number of participants studied was 424.

### Interventions

Participants randomized to each of the three interventions had a face-to-face interview with the dietitian plus a group session. The same dietitian delivered the interventions to the three groups in each center. In the individual session, and based on the individuals' adherence to the MD or to the low-fat diet according to a 14-point score and 9-point score questionnaire, respectively, each participant received personal recommendations for changes to be introduced to their diet in order to achieve the goals of the assigned intervention group during a 30-min session. Positive recommendations for increasing the consumption of vegetables, fruits, legumes, fish and seafood, and white meats, were given to each group. Negative recommendations included limiting the consumption of red and processed meats, fat-rich dairy products, sweets and pastries, snacks and sweetened beverages. However, recommendations for total fat intake differed between the two MD groups and the low-fat diet groups. Therefore, whereas participants in the low-fat group were advised to reduce all types of fat, recommendations for the MD groups included increasing the intake of vegetable fats and oil. No energy restrictions were suggested for any of the intervention groups. Group sessions (up to 20 participants) were organized separately for each of the three intervention groups. The dietitian provided informative talks and written material, with descriptions of target foods, seasonal shopping lists, meal plans, and recipes. Participants in the MD groups were given a free 3-month supply of VOO (1 L/wk) or mixed nuts (30 g/d, as 15 g walnuts, 7.5 g hazelnuts and 7.5 g almonds). The individual and group visits were repeated every 3 months with the same content, with the exception that shopping lists and recipes varied with the season of the year. All participants had free and continuous access to their center's dietitian throughout the study.

### Measurements

At baseline participants were administered several questionnaires [23], [24]: (a) a 14-item questionnaire assessing the degree of adherence to the MD (values of 0 to 1 were assigned to each item, so that a score of 14 points meant the maximum adherence); this was an extension of a previously validated questionnaire; (b) a general 47-item questionnaire about education, lifestyle, medical conditions, and medication use; (c) a 137-item food frequency questionnaire (FFQ) adapted from a previously validated FFQ; and (d) a validated Spanish version of the Minnesota Leisure-Time Physical Activity Questionnaire. Moreover, participants underwent anthropometric and BP measurements and collection of fasting blood samples. All examinations were repeated at 1 year, with the exception of the general questionnaire, which was substituted by a follow-up questionnaire.

### Anthropometry

The anthropometric measures used in this study were height (m), weight (kg), body mass index (BMI, calculated as weight in kg/height^2^ in m^2^) and waist circumference (WC). Height and weight (with light clothing and no shoes) were recorded using a calibrated balance beam scale and a wall-mounted calibrated stadiometer, respectively. Waist circumference was measured using an anthropometric measuring tape, at a horizontal plane midway between the lowest rib and the iliac crest. Blood pressure was measured in triplicate with a validated semi-automatic sphygmomanometer after a minimum of 5 min rest in the seated position.

### Laboratory measurements

Blood samples were collected after an overnight fast, coded, shipped to a central laboratory, and stored at −80°C until analyses. Laboratory technicians were blinded to the intervention. Plasma glucose level was analyzed using the glucose-oxidase method; total serum cholesterol (TC) and triglyceride (TG) levels were measured using enzymatic procedures; and high-density lipoprotein cholesterol (HDL-C) level was determined after precipitation with phosphotungstic acid and magnesium chloride [24]. The plasma FA profile was determined by fast gas chromatography with a previous derivatization to their corresponding fatty acid methyl esters [25]. Results were expressed as relative percentages of total FA. The average of two measures was used for the analysis of laboratory variables both at baseline and 1 year.

### Metabolic syndrome definition

The recent definition of MetS proposed by six major organizations and societies (IDF, NHLBI, AHA, WHF, IAC, and IASO) [1] was used in the present study, such that the presence of any three of the five following risk factors constitutes a diagnosis of MetS: elevated WC, elevated TG (≥150 mg/dL), reduced HDL-C (<40 mg/dL in men and <50 mg/dL in women), elevated BP (SBP ≥130 mmHg and/or DBP ≥85 mmHg), and elevated fasting glucose (≥100 mg/dL). Drug treatment for these specific conditions is an alternative indicator. In this definition, it is possible to use either the IDF or the AHA/NHLBI cut-off limits for WC (≥94 cm for men and ≥80 cm for women, or >102 cm for men and >88 cm for women, respectively) in European populations. The latter was used in the present study.

### Statistical analysis

All values are presented as mean ± SD, unless otherwise indicated. Kolmogorov-Smirnov tests were carried out to check variable distribution. Since the statistical distribution of plasma FA concentrations was found to be skewed, geometric means were used to describe FA concentrations. Moreover, FA concentrations were log-transformed for analysis to improve normality. Analysis of variance (ANOVA) and the χ^2^ test were used to determine differences in baseline characteristics in individuals with and without MetS for continuous and categorical variables, respectively. Besides the assignation to one MD group, participants were divided into four MetS groups: those who did not change their MetS status after the 1-year intervention (always/never) and those whose status changed (reversion/incidence). Within- and between-group changes in plasma FA concentrations were examined with paired *t* tests and ANOVA followed by the Duncan test, respectively. The relationship between these changes and 1-year changes in metabolic risk factors was determined by partial correlation analysis controlling for gender, age, smoking status, occupation, educational level, baseline energy intake, 1-year changes in energy intake, and medication for hypercholesterolemia, BP, and diabetes. To examine the associations between these changes and the incidence and reversion of MetS, participants were categorized by quartiles of 1-year changes in plasma concentrations of FA. Then, a logistic regression analysis was carried out to calculate the odds ratios (OR) and 95% confidence intervals (CI) of the incidence and reversion of MetS according to these quartiles. Finally, we used McNemar's test and logistic regression models to assess within- and between-quartile changes in the prevalence of MetS. All logistic regression models were adjusted for potential confounding factors (age, gender, baseline MetS status, baseline overweight/obesity, and 1-year changes in BMI), and the lowest quartile was used as the reference. For all analyses, two-sided significance was determined at *p*<0.05. Analyses were performed using SPSS version 15.0 (SPSS Inc., Chicago, IL, USA).

## Results

The baseline characteristics of the 424 participants (175 men and 249 women) that completed the intervention program at 1 year are detailed in Table 1 according to their MetS status. By design, participants were mostly overweight subjects with an elevated number of CVD risk factors. Of the total population, 92.7%, 84.2%, 68.4%, and 45.8% were overweight or obese, hypertensive, dyslipidemic, or had type II diabetes, respectively. The prevalence of MetS in the study population was 67.7%, whereas that of its components was 96.0%, 64.6%, 63.8%, 58.5%, and 55.7%, for elevated BP, WC, fasting glucose, TG, and reduced HDL-C, respectively. As expected, most of the characteristics associated with MetS were significantly higher among those who presented with this condition.

**Table 1:** Baseline characteristics of participants completing 1-year intervention.^A^

Characteristics	No MetS (*n* = 137)	MetS (*n* = 287)	*p* B
Age (years)	67.5±6.1	67.7±5.8	0.833
Men (%)	52.6	35.9	0.001
Weight (kg)	71.5±9.8	75.5±10.4	<0.001
BMI (kg/m^2^)	27.9±3.0	30.0±3.1	<0.001
Overweight or obese (%)^C^	86.1	95.8	<0.001
Hypertension (%)^D^	84.7	84.0	0.854
Dyslipidemia (%)^E^	56.9	73.9	<0.001
Type 2 diabetes mellitus (%)	33.6	51.6	0.001
Family history of premature CHD (%)^F^	21.9	19.5	0.805
Current smoker (%)	18.2	12.5	0.118
MetS components (%)^G^			
Elevated WC	29.9	81.2	<0.001
Elevated TG	21.2	76.3	<0.001
Reduced HDL-C	19.7	72.8	<0.001
Elevated BP	90.5	98.6	<0.001
Elevated fasting glucose	36.5	76.9	<0.001
Medications (%)			
Aspirin or antiplatelet drugs	21.2	24.4	0.593
Antihypertensive agents	67.9	75.3	0.110
Hipolypidemic agents	19.0	56.4	<0.001
Insulin	6.6	6.3	0.783
Hypoglycemic agents	16.8	34.8	<0.001
Occupation (%)			
Worker	16.1	10.2	0.078
Unemployed or unfit	24.1	31.2	0.141
Retired	59.9	58.6	0.745
Education level (%)			
None	2.2	4.6	0.237
Primary school	64.4	72.3	0.116
Secondary school	25.9	15.6	0.012
University	7.4	7.4	0.995

MetS, Metabolic Syndrome; BMI, body mass index; CHD, coronary heart disease; WC, waist circumference; TG, triglycerides; HDL-C, high density lipoprotein cholesterol; BP, blood pressure.

A Values are expressed as mean ± SD or percentage of participants.

B *p* value for comparison between groups calculated by one-factor ANOVA for continuous variables or χ^2^ test for categorical variables.

C BMI≥25 kg/m^2^.

D Systolic BP≥140 mmHg or diastolic BP≥90 mmHg or antihypertensive medication.

E LDL cholesterol ≥160 mg/dL or lipid-lowering therapy; HDL cholesterol ≤40 mg/dL in men or ≤50 mg/dL in women.

F Definite myocardial infarction or sudden death before 55 years in male first-degree relatives or before 65 years in female first-degree relatives.

G The metabolic syndrome components are defined according to the IDF, NHLBI, AHA, WHF, IAC, and IASO recent criteria.

Table 2 shows the baseline plasma FA profile and 1-year changes according to the intervention group. Participants in the MD+VOO group showed significantly increased plasma concentrations of palmitic acid (PA, C16:0) and oleic acid (OA, C18:1*n*-9) but significantly reduced proportions of margaric acid (MGA, C17:0), stearic acid (SA, C18:0), and linoleic acid (LA, C18:2*n*-6). In contrast, subjects in the MD+nuts group showed a significant increase in the levels of PA, LA, and α-linolenic acid (ALA, C18:3*n*-3), but significantly reduced proportions of myristic acid (MA, C14:0), MGA, palmitoleic acid (POA, C16:1*n*-7), and dihommo-γ-linoleic acid (DGLA, C20:3*n*-6). Finally, those in the control group only showed a significant increase in the plasma levels of PA with a reduction in MGA and ALA after 1 year of intervention. Between-group differences were only significant for some MUFA (OA) and PUFA (LA, DGLA, and ALA).

**Table 2:** Plasma fatty acid levels at baseline and 1-year changes according to the intervention groups.^A^

Fatty acid	MD+VOO (*n* = 142)	*p* B	MD+Nuts (*n* = 141)	*p* B	Control (*n* = 141)	*p* B	*p* C
***SFA***							
**MA (C14:0)**							
Baseline	0.57±0.27		0.63±0.31		0.62±0.27		
Change	0.012^a^±0.28	0.573	−0.060^b^±0.33	0.008	−0.0087^a,b^±0.33	0.714	0.067
**PA (C16:0)**							
Baseline	21.55±2.20		21.37±2.06		21.96±2.45		
Change	0.51±2.00	0.002	0.37±1.98	0.024	0.67±2.57	0.001	0.517
**MGA (C17:0)**							
Baseline	0.27±0.34		0.30±0.35		0.28±0.33		
Change	−0.030±0.34	0.034	−0.037±0.42	0.048	−0.031±0.38	0.036	0.981
**SA (C18:0)**							
Baseline	6.76±1.16		6.90±1.09		6.74±1.26		
Change	−0.25±1.23	0.005	−0.15±1.20	0.101	−0.055±1.46	0.604	0.325
***MUFA***							
**POA (C16:1** ***n*** **-7)**							
Baseline	1.35±0.69		1.33±0.57		1.36±0.59		
Change	−0.012±0.49	0.766	−0.093±0.55	0.033	−0.034±0.58	0.410	0.221
**OA (C18:1** ***n*** **-9)**							
Baseline	25.29±4.45		26.42±3.98		26.75±4.48		
Change	2.62^a^±3.42	<0.001	−0.061^b^±3.34	0.830	−0.24^b^±4.40	0.520	<0.001
***PUFA***							
***Series n-6 FA***							
**LA (C18:2** ***n*** **-6)**							
Baseline	29.47±5.42		28.09±4.72		28.00±4.98		
Change	−1.94^a^±4.26	<0.001	1.81^b^±3.92	<0.001	0.82^b^±5.89	0.086	<0.001
**GLA (C18:3** ***n*** **-6)**							
Baseline	0.39±0.18		0.40±0.20		0.41±0.18		
Change	0.013±0.20	0.536	−0.0027±0.15	0.841	0.019±0.20	0.264	0.667
**DGLA (C20:3** ***n*** **-6)**							
Baseline	1.50±0.35		1.50±0.36		1.48±0.34		
Change	−0.040^a,b^±0.32	0.132	−0.077^a^±0.24	<0.001	0.030^b^±0.37	0.294	0.010
**AA (C20:4** ***n*** **-6)**							
Baseline	6.75±1.74		6.72±1.63		6.64±1.75		
Change	−0.22±1.23	0.057	−0.080±1.21	0.408	0.081±1.59	0.536	0.160
***Series n-3 FA***							
**ALA (C18:3** ***n*** **-3)**							
Baseline	0.33±0.16		0.32±0.12		0.33±0.15		
Change	−0.021^a^±0.18	0.101	0.15^b^±0.19	<0.001	−0.032^a^±0.19	0.020	0.028
**EPA (C20:5** ***n*** **-3)**							
Baseline	0.74±0.82		0.73±0.54		0.71±0.65		
Change	0.055^a^±0.78	0.196	0.084^a^±0.60	0.062	−0.065^b^±0.67	0.067	0.060
**DHA (C22:6** ***n*** **-3)**							
Baseline	2.51±0.95		2.48±0.81		2.43±0.69		
Change	0.012^a,b^±1.25	0.899	−0.081^a^±0.83	0.215	0.18^b^±1.07	0.068	0.073

MetS, Metabolic Syndrome; MA, myristic acid; PA, palmitic acid; MGA, margaric acid; SA, stearic acid; POA, palmitoleic acid; OA, oleic acid; LA, linoleic acid; GLA, *γ*-linolenic acid; DGLA, dihommo-γ-linoleic acid; AA, arachidonic acid; ALA, *α*-linolenic acid; EPA, eicosapentaenoic acid; DHA, docosahexaenoic acid.

Values in the same row (Change) with different superscript letters (a,b) are significantly different (*p*<0.05 by Duncan test).

A Values are expressed as geometric mean (% of total fatty acids) ± SD.

B *p* for within-group differences from baseline by paired *t* test.

C *p* for between-group differences from baseline by one-factor ANOVA.

The baseline plasma FA profile and 1-year changes according to the 1-year change in MetS status are shown in Table 3. Participants who never had MetS showed significantly higher proportions of PA, OA, and ALA, but reduced proportions of DGLA after 1 year of intervention. In turn, participants in the MetS reversion group showed significantly increased plasma levels of OA and ALA, whereas those in the MetS incidence group showed significantly increased plasma concentrations of MA and PA, but presented a reduction in the levels of MGA and ALA. Finally, participants that always had MetS during the intervention showed a significant increase in the proportions of PA and OA with a significant reduction in the plasma concentrations of MGA and SA. Between-group changes in FA concentrations were significantly different only in the case of OA.

**Table 3:** Plasma fatty acid levels at baseline and 1-year changes according to the 1-year change in the MetS status.^A^

Fatty acid	Never (*n* = 97)	*p* B	Reversion (*n* = 44)	*p* B	Incidence (*n* = 40)	*p* B	Always (*n* = 243)	*p* B	*p* C
***SFA***									
**MA (C14:0)**									
Baseline	0.53±0.21		0.58±0.31		0.56±0.26		0.65±0.30		
Change	−0.012^a^±0.22	0.552	−0.048^a^±0.34	0.212	0.089^b^±0.30	0.024	−0.034^a^±0.34	0.085	0.057
**PA (C16:0)**									
Baseline	20.70±1.86		21.77±2.66		21.49±2.24		22.00±2.22		
Change	0.58^a,b^±2.06	0.003	0.16^a^±2.65	0.666	0.98^b^±1.81	0.001	0.47^a,b^±2.22	0.001	0.314
**MGA (C17:0)**									
Baseline	0.27±0.28		0.26±0.31		0.30±0.34		0.29±0.36		
Change	−0.021±0.26	0.224	−0.039±0.32	0.088	−0.072±0.36	0.007	−0.029±0.44	0.028	0.428
**SA (C18:0)**									
Baseline	6.83±1.24		6.83±0.96		6.73±0.79		6.79±1.23		
Change	−0.073±1.40	0.535	−0.25±1.01	0.087	−0.19±0.80	0.136	−0.16±1.37	0.036	0.837
***MUFA***									
**POA (C16:1** ***n*** **-7)**									
Baseline	1.18±0.69		1.35±0.60		1.31±0.58		1.43±0.59		
Change	−0.067±0.51	0.147	−0.067±0.51	0.317	−0.00029±0.45	0.997	−0.034±0.57	0.296	0.783
**OA (C18:1** ***n*** **-9)**									
Baseline	24.96±4.02		25.16±4.82		26.31±4.20		26.79±4.29		
Change	1.12^a,b^±3.71	0.003	2.09^b^±4.32	0.003	−0.72^c^±3.49	0.219	0.63^a,c^±3.97	0.014	0.007
***PUFA***									
***Series n-6 FA***									
**LA (C18:2** ***n*** **-6)**									
Baseline	29.79±4.65		28.47±5.42		29.86±3.69		27.82±5.30		
Change	0.78±4.46	0.088	1.14±6.30	0.215	−0.31±3.51	0.582	−0.064±5.19	0.844	0.290
**GLA (C18:3** ***n*** **-6)**									
Baseline	0.39±0.18		0.41±0.14		0.40±0.22		0.40±0.19		
Change	−0.018±0.16	0.306	0.0078±0.19	0.726	0.014±0.20	0.643	0.021±0.19	0.145	0.437
**DGLA (C20:3** ***n*** **-6)**									
Baseline	1.48±0.34		1.48±0.36		1.53±0.36		1.49±0.35		
Change	−0.076±0.30	0.010	0.048±0.42	0.410	−0.044±0.30	0.306	−0.022±0.30	0.253	0.134
**AA (C20:4** ***n*** **-6)**									
Baseline	6.98±1.64		6.52±1.59		6.74±1.71		6.62±1.75		
Change	−0.24^a^±1.27	0.056	0.38^b^±1.21	0.050	−0.35^a^±1.31	0.063	−0.044^a,b^±1.41	0.622	0.036
***Series n-3 FA***									
**ALA (C18:3** ***n*** **-3)**									
Baseline	0.32±0.14		0.29±0.15		0.35±0.14		0.33±0.15		
Change	0.041^a^±0.20	0.010	0.087^a^±0.23	0.008	−0.061^b^±0.19	0.025	0.021^a^±0.21	0.052	0.009
**EPA (C20:5** ***n*** **-3)**									
Baseline	0.84±0.66		0.75±0.80		0.63±0.34		0.70±0.71		
Change	0.036±0.73	0.484	0.027±0.96	0.757	0.002±0.41	0.969	0.018±0.66	0.506	0.988
**DHA (C22:6 ** ***n*** **-3)**									
Baseline	2.61±0.81		2.58±0.93		2.35±0.59		2.42±0.64		
Change	0.019±1.06	0.844	0.054±1.08	0.717	0.11±0.97	0.400	0.024±1.09	0.703	0.953

MetS, Metabolic Syndrome; MA, myristic acid; PA, palmitic acid; MGA, margaric acid; SA, stearic acid; POA, palmitoleic acid; OA, oleic acid; LA, linoleic acid; GLA, *γ*-linolenic acid; DGLA, dihommo-γ-linoleic acid; AA, arachidonic acid; ALA, *α*-linolenic acid; EPA, eicosapentaenoic acid; DHA, docosahexaenoic acid.

Values in the same row (Change) with different superscript letters (a,b) are significantly different (*p*<0.05 by Duncan test).

A Values are expressed as geometric mean (% of total fatty acids) ± SD.

B *p* for within-group differences from baseline by paired *t* test.

C *p* for between-group differences from baseline by one-factor ANOVA.

After 1 year of intervention, changes in several specific FA were related to changes in various metabolic risk factors (Table 4). When significant, changes in most SFA and POA were adversely associated with changes in metabolic risk factors, whereas changes in most PUFA were associated in a beneficial way. In addition, we also calculated partial correlation coefficients to look for possible relationships between food groups and plasma FA composition. Since plasma concentrations of OA, LA, and ALA are good biomarkers of olive oil, seed oil, and walnut consumption, the 1-year changes reported indicate good adherence to the food supplied. The increase in plasma levels of OA and ALA in the MD+VOO and MD+nuts groups indicates good adherence to OO and nuts, respectively, whereas the decrease in the concentration of LA and the clear inverse relation found between OO consumption and *n*-6 PUFA levels suggests that people in the MD+VOO group replaced seed oils with VOO.

**Table 4:** Partial correlation coefficients between 1-year changes in plasma fatty acids and 1-year changes in metabolic risk factors (*n* = 424) and between food groups and plasma fatty acids.^A^

Fatty acid	WC	BMI	TC	HDL-C^B^	LDL-C	TG^B^	SBP	DBP	glucose	Food groups
***SFA***										
MA (C14:0)	0.02	0.00	0.13*	0.01	0.10*	0.10*	0.06	0.03	0.06	cheese (0.10*)
PA (C16:0)	0.00	0.01	0.09	−0.01	0.02	0.17*	0.06	−0.01	0.07	
MGA (C17:0)	−0.06	−0.01	0.04	−0.12*	0.10	−0.04	−0.07	−0.09	−0.03	milk (0.11*)
SA (C18:0)	0.06	−0.02	−0.02	0.01	0.03	−0.13*	−0.06	−0.03	0.00	
***MUFA***										
POA (C16:1*n*-7)	0.03	0.14**	0.06	−0.02	0.00	0.21***	0.00	0.02	0.06	
OA (C18:1*n*-9)	−0.02	0.09	−0.03	0.02	−0.07	0.03	0.03	−0.03	−0.03	OO (0.17***), seed oils (−0.18***)
***PUFA***										
***Series n-6***										
LA (c18:2*n*-6)	−0.03	−0.06	0.04	0.02	0.07	−0.20**	−0.02	0.02	0.01	OO (−0.19***) seed oils (0.19***), total nuts (0.11*)
GLA (C18:3*n*-6)	−0.01	−0.02	0.05	−0.08	0.05	0.08	−0.04	0.03	0.01	
DGLA (C20:3*n*-6)	−0.08	0.05	0.01	0.03	0.04	−0.08	0.01	0.03	−0.14**	
AA (C20:4*n*-6)	−0.05	−0.04	−0.19**	0.02	−0.08	−0.32***	−0.08	−0.11*	−0.18***	
***Series n-3***										
ALA (C18:3*n*-3)	−0.12*	−0.09	0.05	0.07	0.07	−0.05	−0.06	0.04	−0.07	total nuts (0.10*), walnuts (0.17***)
EPA (C20:5*n*-3)	−0.07	−0.04	0.11*	0.10*	0.13*	−0.09	0.09	0.03	0.02	white fish (0.17***), blue fish (0.29***)
DHA (C22:6*n*-3)	0.02	−0.09	−0.05	−0.04	0.01	−0.13*	−0.05	−0.01	−0.02	white fish (0.15***), blue fish (0.25***)

WC, waist circumference; BMI, body mass index; TC, total cholesterol; HDL-C, high density lipoprotein cholesterol; LDL-C, low density lipoprotein cholesterol, TG; triglycerides; SBP, systolic blood pressure; DBP, diastolic blood pressure; MA, myristic acid; PA, palmitic acid; MGA, margaric acid; SA, stearic acid; POA, palmitoleic acid; OA, oleic acid; LA, linoleic acid; GLA, *γ*-linolenic acid; DGLA, dihommo-γ-linoleic acid; AA, arachidonic acid; ALA, *α*-linolenic acid; EPA, eicosapentaenoic acid; DHA, docosahexaenoic acid; OO, olive oil.

* *p*<0.05,

** *p*<0.01,

*** *p*<0.001.

A Adjusted for sex, age, smoking status, occupation, educational level, baseline energy intake, 1-year changes in energy intake, and medication for hypercholesterolemia, blood pressure, and diabetes.

B Log-transformed for normality.

**Figure 2:**
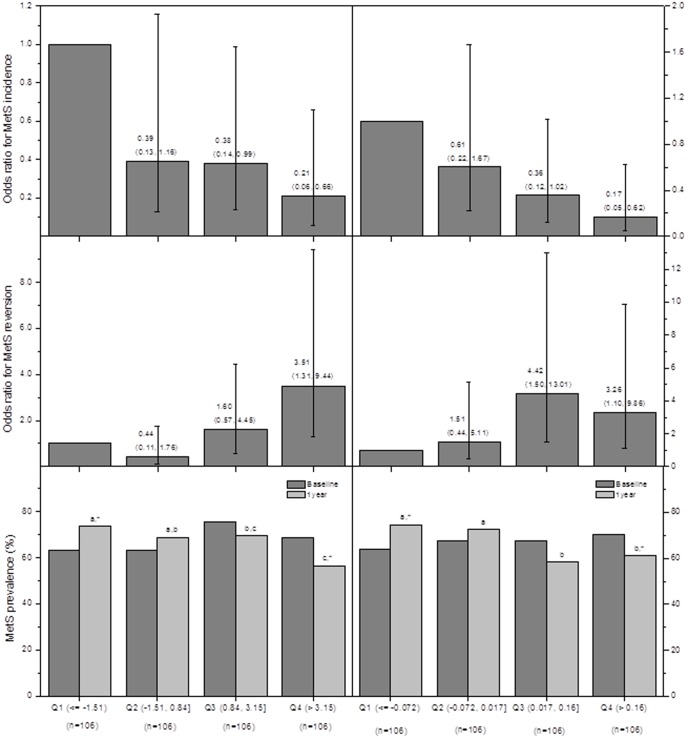
Odds ratios (95% confidence intervals) of 1-year MetS incidence and reversion, and MetS prevalence. Odds ratios at baseline and 1 year by quartiles of 1-year changes in plasma levels of oleic and α-linolenic acids are shown in the left and right panels, respectively. The lowest quartile was chosen as the reference for the odds ratio calculations. Different superscript letters indicate significantly different 1-year prevalence changes between quartiles (*p*<0.05 by logistic regression model), while an * indicates a significant 1-year prevalence change within each quartile (*p*<0.05 by McNemar's test).

In logistic regression analyses, higher changes in the proportions of MA and PA were related to an increased incidence of MetS (*p* between quartiles quartiles = 0.037 and 0.046, respectively), with an OR (95% CI) for the 1^st^, 2^nd^, 3^rd^, and 4^th^ quartiles of changes of 1.00, 1.90 (0.44, 8.11), 2.70 (0.65, 11.24), and 6.43 (1.53, 27.03), and 1.00, 2.17 (0.46, 10.3), 4.06 (0.96, 21.20), and 8.65 (1.79, 41.78), respectively. One the other hand, both biomarkers of the foods supplied, OA and ALA, were found to be protective against MetS. Hence, changes in OA and ALA concentrations were inversely associated with the incidence of MetS (*p* between quartiles = 0.035 and 0.032, respectively) and showed a stronger direct relation with MetS reversion (*p* between quartiles = 0.005 and 0.020, respectively) (Figure 2). No other significant associations were found between changes in the levels of the remaining FA and the incidence or reversion of MetS. Finally, changes in the prevalence of MetS according to the quartiles of change in OA and ALA were 10.4%, 5.7%, −5.7%, and −12.3% (*p* between quartiles = 0.002), and 10.4%, 4.7%, −9.4%, and −9.4% (*p* between quartiles = 0.002), respectively. Moreover, no significant weight changes were observed across the quartiles of changes in OA and ALA (*p* between quartiles = 0.931 and 0.745, respectively).

## Discussion

To our knowledge, the present study is the first randomized controlled trial to have assessed the effect of a Mediterranean dietary pattern on the overall plasma FA profile of individuals at high risk of CVD and its relation with MetS. Our results show a robust association between two MD and changes in the FA composition after 1 year of intervention.

Participants in the MD+VOO group showed reduced plasma levels of MGA, SA, and LA but increased levels of PA and OA. The findings in the MD+VOO group (40% and 20.5% of energy from fats and MUFA, respectively) are consistent with those of other intervention studies using MUFA-enriched diets high in fat content and with a similar nutrient composition [26], [27]. This is important since total fat intake modifies the plasma FA composition [28]. In both studies, people receiving a MUFA diet (40%/20% and 39%/20.3% of energy from fats/MUFA, respectively) showed increased levels of PA and decreased levels of SA, although these changes were only significant in the former study; POA levels did not change significantly. In addition, the LA concentrations were significantly reduced with an increase in OA due to supplementation. In turn, the MD+nuts group showed reduced concentrations of MA, MGA, POA, and DGLA, while increasing the levels of LA and ALA. These results are consistent with a recent controlled dietary intervention that reported significantly lower serum proportions of PA, SA, POA, and DGLA, but significantly higher proportions of LA and ALA in slightly overweight and hyperlipidemic healthy subjects that consumed a rapeseed oil-based diet rich in MUFA and PUFA (particularly OA, LA, and ALA) compared to a diet high in saturated fat [15]. Interestingly, the MD+nuts group diet induced a FA composition that was beneficial in the face of cardiometabolic disease, since high proportions of PA, POA, and DGLA, and reduced levels of LA have been related to several cardiometabolic diseases [18]–[21], including MetS [22]. Consistently, we also found that increases in individual SFA and POA correlated adversely with several metabolic risk factors.

We also examined the association between the adherence to a MD and the incidence, reversion, and changes in prevalence of MetS. Rather than performing analyses according to the intervention group, we used biomarkers of the foods supplied to each group for two reasons. First, as inhabitants of a Mediterranean region, the participants in the control group consumed a lower amount of fatty foods characteristic of the Western diet but not a lower amount of VOO or nuts, which could have attenuated any differences between groups. Secondly, since biomarkers are potentially independent of the errors associated with self-report methods [14], they provide an objective and accurate measure of the consumption of MD foods among participants independently of the assigned group. Some FA are considered to be indirect biomarkers of the consumption of individual foods or food groups. This is the case when the food source is the primary source of the FA and it is stable in the sampling medium [14]. According to the correlation analysis, plasma concentrations of OA and ALA were good biomarkers of OO and walnut intake, respectively. Walnuts are characterized by their high ALA content, which, in turn, cannot be synthesized endogenously. Therefore the correlation between ALA in plasma and walnut intake was expected. Regarding OA, being a MUFA, it can be synthesized endogenously from either carbohydrates (CHO) or SFA, particularly when MUFA intakes are lower. Moreover, although OO is the richest dietary source of OA, it is also a major component of most animal fats and makes up a sizable fraction of most vegetable oils. However, while animal fats rich in OA typically contribute considerably to its intake in other European countries, OO was the largest contributor of total fat (70%) in our Catalan population. Moreover, 70–80% of its MUFA content is due to OA. Therefore, OA was a good biomarker of OO consumption in our sample population. In fact, the plasma levels of OA have previously been related to their dietary source in Mediterranean populations [29]. Furthermore, tissue stores of OA were a marker of OO consumption in Spain, but not in the other four European centers of the EURAMIC study [30].

Using the biomarkers of foods supplied to the intervention groups we found that the incidence and reversion rates of MetS progressed inversely and in parallel, respectively, to increases in OA and ALA, which resulted in significant differences in the changes in prevalence of MetS across the quartiles of changes in both OA and ALA. Therefore, the beneficial effect of OO and nut consumption on the prevalence of MetS appears to be a consequence of the combination of incidence and reversion rates. However the reduction in the prevalence of MetS obtained in our study was much lower than that reported by other studies showing a beneficial effect of adherence to the MD in terms of MetS prevalence [9]. This could be due to several factors. Firstly, participants in the other intervention studies were younger and all had MetS or impaired glucose tolerance at baseline. Secondly, the CHO intake in those studies was higher than that of our study population (>55% of energy vs. 40%) and extensive research has achieved consensus that reducing dietary CHO is a critical approach to treating or managing the manifestations of MetS [31]. Finally, energy intake was reduced and physical activity increased in the previous studies so as to achieve weight loss, which is considered the number one priority when treating MetS [6]. However, no restrictions were prescribed in any of our groups, which resulted in a non-significant weight change across the quartiles of changes in OA and ALA. Indeed, a recent study reported that a MD without weight loss led to non-significant changes in the components or prevalence of MetS compared to the control diet, whereas only a small reduction in body weight (10%) reduced the prevalence of MetS by more than 50% [32]. However, the magnitude of the weight loss did not predict the degree of improvement in MetS components while on the MD and patients with the most deteriorated baseline MetS profile presented important cardiometabolic benefits even on a MD without weight loss, suggesting that the MD may achieve beneficial effects even in the absence of weight loss, as was our case.

Apart from the biomarkers of the food supplied, we found that MA and PA were adversely associated with the incidence of MetS. This supports the findings of previous studies about dietary fat quality and disease risk. Hence, substituting unsaturated fat or CHO for SFA impairs insulin sensitivity [33] and induces a FA pattern similar to that observed in people with MetS [17], which is also caused by a diet rich in SFA [15]. Interestingly, we found that when participants were classified according to their change in MetS status, the changes in these two SFA and the two biomarkers of foods supplied evolved in the MetS incidence group in the opposite direction to that of the other groups, with the exception of PA. Thus, while the incidence group showed a significant increase in the proportions of MA and PA and a reduction in the levels of OA and ALA, subjects in the other groups presented a non-significant decrease in the proportions of MA but an increase in the OA and ALA levels. Importantly, although between-group changes in these four FA were not always significant, they differed significantly between the MetS incidence and reversion groups. In fact participants in the reversion group showed the highest reduction in MA, the lowest increase in PA, and the highest increase in both OA and ALA among the non-incident groups, which could explain the change in their MetS status. However, the increase in OA and ALA concentrations observed in participants who always had MetS was probably not enough compared with that of the MetS reversion group to experience a change in their MetS status after the intervention. Furthermore, participants who never had MetS during the intervention probably did not present MetS after 1 year of intervention because they had also significantly increased both OA and ALA.

Interestingly, all these effects were observed without a weight increase despite the high fat content of both MDs. In fact, a meta-analysis of 21 epidemiological studies regarding MD and obesity showed that no studies reported significantly increased obesity in response to a MD. Moreover, over half of the studies provided evidence that adherence to a MD was associated with less overweight/obesity or promoted weight loss [34]. This lack of weight gain and the improvement in overweight or obesity may be due to the increased postprandial fat oxidation, diet-induced thermogenesis and overall daily energy expenditure induced by OO [35] and nut [36] consumption, as well as to a satiating effect [36], [37].

Since the primary endpoint for the treatment of MetS is to reduce the risk of CVD, traditional dietary recommendations such as those proposed by the NCEP, AHA and NHLBI essentially involve following a low-fat diet and achieving weigh reduction by a combination of reduced caloric intake and increased physical activity. In our study, however, we found that a high-fat (>40% of energy) non-energy-restricted diet showed a reduction in MetS prevalence without the need for weight loss or prescribed physical activity. There are several important aspects to consider regarding the MD followed in this study. First of all, it is highly palatable, decreases hunger, and promotes satiety, hence improving long-term adherence compared to low-fat energy-restricted diets [31]. Secondly, no weight gain was observed in participants with high VOO or nut consumption. Finally, a usual consequence of low-fat diets is unrestricted CHO intake, which has been shown to result in reduced HDL-C levels and raised TG levels, thus exacerbating the MetS status [31]. Therefore, the results of this study suggest that there is no rationale for maintaining the fear that a MD rich in fats of vegetable origin may cause weight gain, and that it may be a useful alternative to traditional low-fat diets for the dietary treatment of MetS.

## Supporting Information

Protocol S1 **Predimed Study: Mediterranean diet in the primary prevention of cardiovascular disease.** Amendments to the Research Protocol.
(DOC)

Protocol S2 **Predimed Study: Mediterranean diet in the primary prevention of cardiovascular disease.** Research Protocol. 2009.
(DOC)

Protocol S3 **Predimed Study: Mediterranean diet in the primary prevention of cardiovascular disease.** Research Protocol. 2003.
(DOC)

Checklist S1 **CONSORT checklist.**
(DOC)
