# Supplementary material for: Effects of 1-Year Intervention with a Mediterranean Diet on Plasma Fatty Acid Composition and Metabolic Syndrome in a Population at High Cardiovascular Risk
Source: PLoS One. 2014 Mar 20;9(3):e85202. doi: 10.1371/journal.pone.0085202 (PMC3961210; doi:10.1371/journal.pone.0085202)
Supplement: Protocol S1 — Predimed Study: Mediterranean diet in the primary prevention of cardiovascular disease. Amendments to the Research Protocol. (DOC) [file pone.0085202.s001.doc]

Protocol S1.

###### PREDIMED STUDY

###### MEDITERRANEAN DIET IN THE PRIMARY PREVENTION OF CARDIOVASCULAR DISEASE

###### Amendments to the Research Protocol

Amendments to the Research Protocol

After reviewing the results of the pilot study based on the analysis of the short-term effects of 2 Mediterranean diets versus those of a low-fat control diet on intermediate markers of cardiovascular risk (Ann Intern Med 2006;145:1-11), the Steering Committee decided to perform the following modifications:

1. Oct 2005-One of the initial recruiting centers (Hospital Carlos III, Madrid) abandoned the study after completion of the pilot study. The PI of this center claimed insurmountable logistic difficulties with recruitment to justify his decision.
2. Jan-2006- Another recruiting center (Sevilla University) also abandoned the study in early 2006 after the completion of the pilot study. The PI of this center also claimed insurmountable logistic difficulties with recruitment to justify his decision.
3. Jul 2006- To compensate for the loss of recruiting power, we included two new recruiting centers, thus the number of centers was kept at 11.
4. Oct-2006. Include dietary intervention in the low-fat (control) group.

The PREDIMED group sessions were organized separately for each of the 3 intervention groups. Participants were provided with written material (see: [**http://www.predimed.org**](http://www.predimed.org/) and [**http://www.predimed.es**](http://www.predimed.org/)) including descriptions of seasonal foods, shopping lists, weekly meal plans and cooking recipes. The control group received the same type and intensity of dietary intervention than the two Mediterranean diet groups, although the recommendations for total fat intake were opposite those given to participants in the two Mediterranean diet groups. Advice on vegetables, meats and processed meats, high-fat dairy products, and sweets concurred with the Mediterranean diet, but use of olive oil for cooking and dressing and consumption of nuts and fatty fish were discouraged. A 9-item quantitative score of compliance with the low-fat control diet was constructed (Table 1) as an instrument for dietitians to assess and modify the participant’s dietary pattern but, unlike the 14-item Mediterranean diet score, it was not an intervention outcome. The last assessment of the 9-item score helped dietitians to give personalized advice in order to upgrade it in a similar way than the 14-item Mediterranean diet score was instrumental to enhance the Mediterranean diet in the corresponding intervention groups. Similarly, accomplishments in the previous months were used as support to provide further empowerment and self-reward.

1. March 2007-Inclusion Criteria

In the initial protocol we considered as one risk factor criterion for inclusion a plasma HDL-cholesterol concentration <40 mg/dl without gender specification. Based on newly published definition of gender-specific HDL-cholesterol values for cardiovascular risk assessment, we changed this criterion to HDL-cholesterol ≤40 mg/dl in men and ≤50 mg/dl in women.

1. May 2007- Validation of the dietary assessment tools (questionnaires).

The 137-item Food Frequency Questionnaire (FFQ) used in the Predimed trial included a previously validated FFQ (Martin-Moreno, et al. Int J Epidemiol 1993; 22: 512-9.) plus some additional items which were more specific for our trial. During the course of the study we had the opportunity to repeat the validation study of this FFQ. The validation field work was done during 2005-2007 and the results were eventually published (Fernández-Ballart JD. et al. **Br J Nutr**. 2010 Jun;103(12):1808-16 and de la Fuente-Arrillaga C, et al. **Public Health Nutr**. 2010 Sep;13(9):1364-72).

In addition, we have also published the validation study for the brief 14-item questionnaire of adherence to the Mediterranean Diet used in the trial (Schröder H, et al. **J Nutr**. 2011 Jun;141(6):1140-5).

1. Apr-2008: Change of Sample Size

The sample size was recalculated after the DSMB meeting in April 2008 on the basis of the observed rates of cardiovascular events during the first 2 years of follow-up. The ALLHAT trial included similar participants and observed an 8.9% cumulative rate for the primary outcome (fatal CHD + non-fatal myocardial infarction) after 4.9 years of follow-up (*JAMA* 2002; 288: 2998-3007). Adapting this figure to a 6-year follow-up and including also stroke in the end-point definition, an 11% absolute risk in the control group could be conservatively assumed in our study. We expected a 25% relative risk reduction in both MeDiet groups. Under these assumptions, the total number of participants required was 5631 (1877 per group) for beta=0.2 and 2-tailed alpha=0.05. We needed to include more than 7400 subjects to allow for both 10% losses during follow-up and a lower incidence than expected.

1. Apr-2008. Completion date of the trial

According to the new sample size calculation, the anticipated completion date of the trial was changed to December, 2011.
