# Supplementary material for: Effects of 1-Year Intervention with a Mediterranean Diet on Plasma Fatty Acid Composition and Metabolic Syndrome in a Population at High Cardiovascular Risk
Source: PLoS One. 2014 Mar 20;9(3):e85202. doi: 10.1371/journal.pone.0085202 (PMC3961210; doi:10.1371/journal.pone.0085202)
Supplement: Protocol S3 — Predimed Study: Mediterranean diet in the primary prevention of cardiovascular disease. Research Protocol. 2003. (DOC) [file pone.0085202.s003.doc]

###### Protocol S3.

###### PREDIMED STUDY.

###### MEDITERRANEAN DIET IN THE PRIMARY PREVENTION OF CARDIOVASCULAR DISEASE

###### Research Protocol

October 2003

###### MEDITERRANEAN DIET IN PRIMARY CARDIOVASCULAR PREVENTION

###### Research Plan

A. SPECIFIC AIMS

A Mediterranean-type diet (MeDiet) has been widely considered as a model of healthy eating. The adherence to a MeDiet was associated with lower coronary mortality in a cohort study (Trichopoulou, 2003) and in a relatively small trial with patients after a myocardial infarction (De Lorgeril, 1999), but no large primary prevention trial with clinical events as end-point has been performed. Furthermore, there are discrepancies among nutrition experts because of the high-fat content of MeDiets (up to >40% of total energy intake), which is in conflict with the usual recommendation to follow a low-fat diet in order to avoid overweight/obesity and to prevent coronary heart disease (CHD) (Ferro-Luzzi, 2003; Jequier, 2002; USDA, 2000; Connor, 1997; Bray, 1998). The potential cardiovascular preventive effect of MeDiets in the face of the huge and increasing global burden of cardiovascular disease (Reddy, 2004; WHO, 2003a) makes the answer to this question a priority for public health. Other important reasons to thoroughly assess the effect of dietary guidelines based on the MeDiet, include: a) the long tradition of following MeDiets without any harm in Southern Europe, where life expectancy is extraordinarily high (Willett, 1995), b) the low incidence of CHD in these countries in spite of having similar or even higher levels of classical risk factors compared to the US population (Tunstall-Pedoe, 1999); c) the diversity of mechanistic and epidemiological observations of beneficial effects on cardiovascular health of the consumption of key components of the MeDiet, such as monounsaturated fatty acids (MUFA), extra-virgin olive oil (EVOO), or nuts; and d) the higher palatability, acceptance and compliance with MeDiets in comparison with low-fat diets (McManus, 2001).

In the era of evidence-based medicine, nutritional recommendations should be based in large scale randomized intervention studies in which clinically relevant (“hard”) end-points are evaluated. However, no randomized controlled trial has ever been conducted to assess to what extent a MeDiet offers greater benefits than a usual diet in the primary prevention of cardiovascular events. This is the research plan of the first primary prevention trial, the PREDIMED Study **(PREDIMED** meaning ***PREvención con DIeta MEDiterránea*),** which plan to test the effects of a MeDiet intervention on prevention of cardiovascular disease. The results of this large scale randomized trial should provide the strong evidence required to support dietary guidelines for the general public. This study needs to be conducted in Spain for two reasons: a) It is more cost-effective to expand an ongoing trial that has developed all its infra-structure than to start a new one; b) the US population is not yet prepared to adopt a MeDiet in a short period of time.

A.1 Primary Specific Aims

Specific Aim 1 (MeDiet): to test the effect of a MeDiet on a composite endpoint of cardiovascular death, myocardial infarction, coronary revascularization procedures, and stroke.

Specific Aim 2 (MeDiet+Nuts): to test the effect of a MeDiet enriched with supplemental walnuts, almonds, and hazelnuts on a composite endpoint of cardiovascular death, myocardial infarction, coronary revascularization procedures, and ischemic stroke

A.2 Other Specific Aims

We will ascertain changes in blood lipids, blood pressure (BP), markers of inflammation and other intermediate markers of cardiovascular risk to better understand how dietary changes are able to modify the risk of clinical events.

B. BACKGROUND AND SIGNIFICANCE

Cardiovascular disease is the main cause of death worldwide at the turn of the XXI century. Western countries, including the US, currently continue to exhibit unacceptable high absolute rates of cardiovascular morbidity and mortality. Furthermore, these diseases constitute emerging and neglected epidemics in developing countries (WHO, 2003a, WHO, 2003b). Surprisingly, a low incidence of CHD is found in some developed countries such as France, Spain, Greece, Italy, and Portugal, leading to a higher life expectancy as compared with Northern European countries or the US (WHO, 1993; Tunstall-Pedoe, 1999). The Mediterranean food pattern has been the factor most frequently invoked to explain this health advantage.

B.1 The Mediterranean diet (MeDiet)

The MeDiet is identified as the traditional dietary pattern found in olive-growing areas of Crete, Greece and Southern Italy in the late 1950s and early 1960s. Its major characteristics are: a) a high consumption of non-refined grains, legumes, nuts, fruits and vegetables; b) a relatively high-fat consumption (even greater than 40 percent of total energy intake) mostly from MUFA, which accounts for 20 percent or more of the total energy intake; c) olive oil used to cook and for dressing salads is the principal source of fat; d) fish consumption is moderate to high; e) poultry and dairy products (usually as yogurt or cheese) are consumed in moderate to small amounts; f) a low consumption of red meats, processed meats or meat products; g) a moderate alcohol intake, usually in the form of red wine consumed with meals (Trichopoulou, 1995).

Replacing saturated fat with MUFA from olive oil produces a decline in total and LDL cholesterol comparable to that attained by low fat diets, but HDL cholesterol is maintained at higher levels, thus obtaining a net advantage on the overall lipid profile (Perez-Jimenez, 2002; Willett, 2000). In addition, LDL particles enriched with oleic acid and with a lesser PUFA content may be less easily converted to modified, proinflammatory LDL particles. High olive oil consumption, the hallmark of MeDiet (Hu, 2003), is partially responsible for these effects. Additional mechanisms have been suggested for the beneficial effect of the MUFA-rich MeDiet (Perez-Jimenez, 2002). Among them (see below), it has been reported that incorporation of oleic acid into cultured endothelial cells decreases the expression of endothelial leukocyte adhesion molecules with reductions in VCAM-1 and inhibition of nuclear factor-kappaB activation (Carluccio, 1999). Postprandial factor VII is significantly lower after a MUFA-rich diet. Olive oil is also associated with a reduced DNA synthesis in human coronary smooth muscle cells (Mata, 1997).

The background of a long and ancient tradition with no evidence of harm makes the MeDiet very promising for public health. The high fruit and vegetable intake adds to its high antioxidant content and other mechanistic benefits provided by the polyphenols present in EVOO and red wine. Wide sectors of the scientific community and of consumers believe in a cardio-protective role for MeDiets. This hypothesis fits well into the current paradigm of studying overall dietary patterns instead of simply assessing isolated nutrients in nutritional epidemiology. The rationale is that food items and nutrients may have synergistic or antagonistic effects when they are consumed in combination. Additionally, overall patterns better represent the dietary practices found in free-living populations and therefore provide more useful epidemiological information (Jacques, 2001; Jacobs, 2003). Consequently, they have a higher potential for acceptability, palatability and future compliance when they are recommended in behavior counseling. Therefore, in spite of its relatively high fat content, the theoretical advantages of the MeDiet are multiple. To increase vegetable consumption, the fat-free or low-fat dressings are less acceptable than the use of olive oil or other full-fat salad dressings. The sautéing or stir frying of vegetables with variable amounts of olive oil instead of using low-fat spreads or steaming increases taste and results in long-term maintenance of a vegetable-rich diet. These preparation and cooking techniques are typical of MeDiet, where the custom is to cook vegetables in olive oil to enhance flavor. Hence, in health promotion and nutritional education a better compliance with MeDiet will be expected. In fact, a recent trial of weight loss (McManus, 2001) reported a better adherence to a MeDiet than to a low-fat diet. Participants viewed this diet as more tasty than low-fat regimens, and this may have been the cause of their increased long-term compliance.

However, at present, there is evidence of an undesirable departure from traditional MeDiets in Southern European countries, especially among younger people (Sanchez-Villegas, 2003; Costacou, 2003), with increasing consumption of red meats, processed meats, and foods and beverages high in simple sugars. In addition, the traditional consumption of EVOO is being replaced by other refined vegetable oils of inferior quality. The presumable antiatherogenic properties of olive oil have been mainly attributed to its high content in MUFA oleic acid. However, in recent years converging evidence indicates that polyphenols only present in EVOO significantly contribute to these benefits. The concentration of phytochemicals in oils is influenced by the oil extraction procedures. EVOO is obtained from the first pressing of the ripe fruit and has a high content in antioxidants (tocopherols, polyphenols, flavonoids) and sterols. Lower quality olive oils (refined olive oils, ROO) lose nearly all of this antioxidant capacity because they are refined by physical and chemical procedures, they are mostly coloreless and aroma free, although their fatty acid composition is close to that of EVOO (Ramirez-Tortosa, 1999; Lercker, 2000). EVOO polyphenolic derivatives (hydroxityrosol, tyrosol and their secoiridoid derivatives) have shown strong antioxidant and anti-inflammatory activity *in vitro* (De la Puerta, 1999; 2001; Manna, 2002; Visioli, 2002). In randomized cross-over trials that compared EVOO with ROO (in doses similar to the usual MeDiet), EVOO increased plasma antioxidant capacity and decreased serum tromboxane B_2_ production. It also increased LDL resistance to oxidation. EVOO affords better protection than dietary alpha-tocopherol against lipid peroxidation (Mataix, 1998) and shows a more comprehensive array of effects related to atherosclerosis reduction, such as reducing oxidative stress (Visioli, 1998). Consequently, ROOs are thought not to provide such high cardiovascular benefits as EVOO and It is important to distinguish between each type of oil when analyzing the effects of olive oil on cardiovascular risk. EVOO has been shown to induce a dramatic regression of atherosclerosis in a hamster model (Mangiapane, 1999). In a randomized trial including 22 hypercholesterolemic men, EVOO improved endothelial function (Fuentes, 2001), and in another trial with 23 hypertensive patients, it significantly reduced BP and the need for antihypertensive medications (Ferrara, 2000). In any case, we assume that the effect of the overall MeDiet pattern will be more important than the isolated action of a single component.

B.2 Available evidence about MeDiet and cardiovascular prevention

A MeDiet was inversely associated with mortality from all-causes in several small observational cohort studies of elderly people (Trichopoulou, 1995; Kouris-Blazos, 1999; Lasheras, 2000). Recent findings from the Greek EPIC cohort, with more than 22,000 participants, suggested that a higher adherence to the MeDiet is associated with a reduction in total mortality and, more specifically, in coronary mortality (Trichopoulou, 2003). Two recent case-control studies also found an inverse association between adherence to the MeDiet and incidence of non-fatal coronary events (Martinez-Gonzalez, 2002; Panagiotakos, 2002). Two secondary prevention trials found a remarkable reduction in reinfarction or death when coronary patients were assigned to a so-called "Mediterranean diet" (De Lorgeril, 1999; Singh, 2002). The first of these trials, the Lyon Diet Heart Study (De Lorgeril, 1999), found a 50%-70% relative reduction in the risk of mortality or reinfarction when patients who had suffered a myocardial infarction were assigned to an experimental diet rich in bread, vegetables, fish, and fruit and low in red meat (replaced with poultry). Butter and cream were replaced with a special margarine rich in alpha-linolenic acid (ALA). The other trial (Singh, 2002) found a 52% relative reduction in total cardiac endpoints with an experimental diet rich in fruits, vegetables, nuts, whole grains and mustard seed or soy bean oil. The 50% to 70% observed reduction of cardiac events in the experimental group of the Lyon trial after 46 months leads us to think that if these results were generalized to non-Mediterranean populations, substantially enhanced and efficient methods to reduce CHD would be available. It would be shortsighted not to acknowledge the vast public health benefit that a MeDiet could provide with adoption by the healthy population-at-large if the findings of the secondary trials are also confirmed in primary prevention trials. The American Heart Association (AHA) has given attention to MeDiets as potentially useful for the prevention of CHD, but a cautious recommendation has been issued highlighting that more studies are needed before advising people to follow a MeDiet. These studies will disentangle whether the diet itself or other factors (such as more physical activity, a beneficial genetic background or stronger social support systems) account for the lower incidence of CHD in the Mediterranean countries (Kris-Etherton, 2001a).

B.3 Limitations of published studies on MeDiet and cardiovascular prevention

The two published trials using so-called “MeDiets” have included patients who had already experienced a clinical event, i.e. they were secondary prevention trials (De Lorgeril, 1999; Singh, 2002), and their results may not be extrapolated to issue dietary guidelines for primary prevention. Additionally, major aspects of the design and methods of these two trials have been overtly criticized. Both trials were designed to provide the experimental group with a high amount of ALA. However, no special consideration was given to olive oil, which is the major source of dietary fat in Mediterranean countries or to the special relevance of EVOO as an important source of polyphenols. On the other hand, no protective effect for olive oil was observed in the Greek EPIC cohort (Trichopoulou, 2003), thus raising the issue of whether the large amount of fat provided by olive oil is in fact affording protection against CHD (Hu, 2003). In the Lyon Diet Heart Study, the assessment of the true dietary patterns and nutrient intakes at baseline and at end of the study were reported for only a subset of participants (30% of the control group and 50% of the experimental group). In addition, no biochemical verification of dietary compliance was done. Thus, the diet followed by most participants completing the trial is not known (Kris-Etherton, 2001a; Robertson, 2001). A third major issue was that only 12.9 percent of energy intake was provided by MUFA in the group assigned to "MeDiet" in the Lyon Trial. In the Indo-Mediterranean Study, the percentage of MUFA intake was even lower (10% of total energy) and the diet of the intervention group (26% fat) could be actually interpreted as a low-fat diet. These values for fat and MUFA intake are far below the higher MUFA content (>=20%) of the traditional MeDiet (Perez-Jimenez, 2002). This is contrary to what should be expected with a MeDiet intervention and these two trials have actually applied interventions not fully in line with the traditional MeDiet. Additional concerns about the Lyon trial are related to the low number of observed end-points (44/14, in the control and intervention group respectively), the improbably large reduction in relative risk (RR) (in spite of not observing changes in some of the classical risk factors), and to the fact that the trial was stopped very early (after 27 months of follow-up). No available randomized trial has assessed an EVOO-rich MeDiet in primary cardiovascular prevention. The PREDIMED study will be the first large trial to randomize high-risk patients to follow-up a traditional MeDiet for primary cardiovascular prevention. The PREDIMED trial is designed to overcome the previous limitations and to provide results with the best quality of evidence.

B.4 Nuts and cardiovascular prevention

Walnuts, almonds, hazelnuts, and other nuts, like pine nuts, are common staples in the traditional MeDiet, since they are locally produced throughout the Mediterranean basin. Nuts are very high in fat (73-90% of the total energy they provide, 48-63 g/100 g edible portion). Most nuts are rich in MUFA (mostly, oleic acid), whereas walnuts are high in PUFAs (linoleic and ALA). The dietary fiber content in nuts is high, ranging from 5 to 9% by weight. Nuts are good sources of arginine, potassium, vitamin E and other bioactive compounds, such as flavonoids, other polyphenols, and phytosterols. Several small randomized trials (<50 subjects) showed consistent decreases in total cholesterol and LDL-cholesterol after interventions with diets high in a variety of nuts, walnuts or almonds (**Kris-Etherton, 2001b**). These results were achieved with intakes that would amount to two or three servings per day. Effects on HDL-cholesterol were inconsistent. When evaluated, the ratios of total cholesterol to HDL cholesterol decreased (**Feldman EB, 2002**). These mechanistic results suggest that frequent nut consumption may decrease the risk of CHD.

A large Californian cohort, the Adventist Health Study, observed that the frequency of nut consumption was inversely associated with cardiovascular risk (**Fraser, 1992**). More recently, three other large cohorts, the Iowa Women's Health Study, the Nurses Health Study and the Physician's Health Study, have consistently shown that frequent nut consumption is associated with a lower CHD risk (**Ellsworth, 2001; Hu, 1998; Albert, 2002**). However, the results of the Iowa cohort did not reach statistical significance and the Physician’s Health Study cohort only found protection for sudden cardiac death, but not for non-sudden coronary death or nonfatal myocardial infarction.

**No available randomized trial has used nuts in primary cardiovascular prevention. The PREDIMED study will be the first large trial** to randomize high-risk patients to receive nuts for cardiovascular prevention.

B.5 High-fat diets based on MUFA in diabetics, overweight subjects and high risk individuals

Traditionally, nutrition advice in diabetics, obese subjects, and those with cardiovascular risk factors emphasized avoiding animal fat and, preferably, all kinds of dietary fat, and replacing them with carbohydrate (CHO). The rationale was that fats provided excess energy, thought to promote obesity. However, scientific evidence has accumulated in the last two decades about the beneficial role of diets with a relatively high MUFA content on cardiovascular risk factors, obesity and diabetes. These beneficial MUFA are provided by the MeDiet and, specifically, by olive oil and most nuts (Ros, 2003; Sabaté, 2003; Garcia-Lorda, 2003). In fact, the frequent intake of simple CHO in many otherwise low-fat foods is associated with weight gain. However, when nutrition advice is given to people with obesity or diabetes, a reluctance still exists to recommend high-fat, high-MUFA diets as an alternative to the traditional (and less palatable) low-fat diets. By the design of the trial, a sizeable number of the PREDIMED participants are either overweight/obese, or type 2 diabetics. It is thus important to recognize that there is no evidence that a higher percentage of fat in the diet in the form of MUFA results in increased body weight. The lack of a fattening effect of such MUFA-rich diets has been shown in the context of controlled diets (Ros, 2003), weight-reduction programs (McManus, 2001), and *ad libitum* diets (Sabaté, 2003). Furthermore, the results of our pilot study (see below) are also sobering with respect to the lack of weight gain in the intervention groups.

B.6. Significance of primary prevention randomized trials

Some contradictory results of observational studies and subsequent large randomized trials have raised major concerns. These conflicting results reinforce the need to obtain the highest available level of evidence before considering any global public health strategy to promote the MeDiet as a model of healthy eating. This evidence is only obtained by conducting large scale randomized trials with clinical cardiovascular events as the principal outcome. Dietary guidelines can be safely issued when consistency is found between observational and experimental studies.

No randomized controlled trial has ever been conducted to assess the MeDiet (or a MeDiet supplemented with nuts) in the primary prevention of cardiovascular disease. While there is a wide consensus that the best answer to the above mentioned questions ideally should be addressed by large randomized trials with “hard” end-points (i.e., clinical events), the difficulties and cost of such trials have precluded their implementation and have led to the belief that they are unfeasible. However, secondary prevention trials using food patterns as main interventions have been conducted with more than 600 participants (De Lorgeril, 1999) or even with 1000 participants (Singh, 2002). We believe that a primary prevention trial is also feasible because we have already recruited (as of July 2004) 2,000 high-risk but disease-free participants for the PREDIMED trial and found evidence after a 3-month follow-up that our behavioral intervention does modify the participants’ dietary pattern and cardiovascular risk profile in the expected direction. An experimental design in the Mediterranean population conducting an intervention only on diet and not on other lifestyle factors will provide the answer to the question formulated by the AHA about the specific protection of the MeDiet on CHD.

C. PRELIMINARY STUDIES

C. 1 Preliminary trials of olive oil and nuts

Among other relevant published trials, we highlight two very recent trials performed by members of our research team.

C. 1.1 Phenolic content in dietary EVOO decreases in vivo LDL oxidation

A randomized double blinded, crossover feeding trial using three similar olive oils with increasing phenolic concentration was conducted in 30 healthy volunteers. Olive oils were administered over three periods of 3 weeks preceded by two-week washout periods. In vivo plasma oxidized LDL exhibited a 34 % reduction (p=0.01) with increasing phenolic content. In vitro resistance of LDL to oxidation also increased from 114 to 122 min (p=0.006). These results supported the hypothesis that EVOO consumption prevents LDL oxidation and could thus reduce the risk of CHD.

C. 1.2 A walnut diet improves endothelial function in hypercholesterolemic subjects

In a crossover design, 21 hypercholesterolemic men and women were randomized to a cholesterol-lowering MeDiet and a diet of similar energy and fat content in which walnuts replaced about 32% of the energy from MUFA. Participants followed each diet for 4 weeks. The walnut diet improved endothelium-dependent vasodilatation and reduced levels of VCAM-1 (p<0.05 for both). The walnut diet significantly reduced total and LDL cholesterol (p<0.05 for both). A cardioprotective effect of nut intake beyond cholesterol lowering was shown suggesting that a walnut-enriched MeDiet may provide even further benefits for cardiovascular prevention.

C. 2 Preliminary case-control study of MeDiet and the risk of myocardial infarction

Using a case-control design (171 cases of myocardial infarction and 171 age-, gender- and hospital-matched controls) we assessed the protection against CHD afforded by a MeDiet. Six food items were considered protective: 1) olive oil, 2) fiber, 3) fruits, 4) vegetables, 5) fish and 6) alcohol. A positive score of 1 to 5 corresponding to the quintile of intake of each of these items was assigned to each participant, while a negative score was assigned for consumption of food groups assumed to be harmful: 7) meat/meat products, and 8) some carbohydrate-rich items with high glycemic index (white bread and other items). The 8 quintile values were summed up for each participant to build a MeDiet score. The higher was the MeDiet score, the lower the odds ratio (OR) of myocardial infarction. A significant linear trend was apparent after adjustment for the main cardiovascular risk factors. For each additional point in the score (observed range: 9–38) the OR (95% confidence interval, CI) was 0.92 (0.86–0.98). Our data supported the hypothesis that increasing compliance with a MeDiet can be an effective approach for reducing the risk of CHD (Martinez-Gonzalez, 2002).

D. DESIGN AND METHODS

D.1 Summary

The proposed study is a parallel group, multi-center, randomized, single-blinded trial aimed at assessing the effects on the risk of major cardiovascular events of two intensive behavioral counseling and nutrition education interventions in comparison with a control group. Both intervention groups are assigned a traditional Mediterranean diet (MeDiet). In one of these two groups we are supplementing the traditional MeDiet with nuts and participants randomized to this group (MeDiet+nuts) receive a free supply of 30 g/d of nuts (15 g walnuts, 7.5 g hazelnuts, and 7.5 g almonds). The third arm of randomization is the control diet group, whose participants do not receive education on the MeDiet, but are given advice on how to follow a low-fat diet. We are recruiting men (age: 55 to 80 years) and women (age: 60 to 80 years) with either diabetes or three or more major cardiovascular risk factors (smoking, high blood pressure (HBP), high LDL cholesterol, low HDL cholesterol, overweight or obesity, and family history of premature CHD). All participants are free of cardiovascular disease at baseline. Study participants are randomized to three equally sized groups (3,500 in each of the three groups). They will be followed-up for clinical outcomes during a median time of four years by the primary care physicians who recruited them for the study. The primary endpoint will be a composite outcome of cardiovascular events, including any of the following: cardiovascular death, myocardial infarction, coronary revascularization procedures, and ischemic stroke.

D. 2 Timeline, progress and need of additional funding for an expanded follow-up.

The Spanish Ministry of Health has funded the current project for the period 2003-2005. The planning of the project took place after receiving the Spanish grant in March 2003 (PI: Ramon Estruch, Funding agency: Fondo de Investigaciones Sanitarias, FIS, Instituto de Salud Carlos III, G03/140). A thematic network of 16 Spanish research groups was then established.

D. 2.1 Planning

Our network hired, trained and certified the needed personnel (a dietitian and a nurse for each center) in all FC. From March to September 2003 we developed the logistics, protocols, operations’ manual, instruments, forms, and data entry/management systems. Each FC contacted approximately 20 Primary Health Care Centers to recruit participants. During this same period, menus and buying lists of Mediterranean products to be provided to participants were developed. Recruitment planning was also completed in eight of the eleven FC. From April to September 2003, dietitians and nurses were trained and certified in nutritional education and procedures for extraction and storage of blood and other biological samples, respectively.

D. 2.2. Implementation

After obtaining reassuring data confirming adequate changes in diet and risk factors during the pilot phase, the recruitment of participants has progressed and is currently ongoing at a good rate. The expertise of Primary Care physicians in clinical trials is enough because most of them had participated previously in other trials. Participants will be recruited in cohorts. Each FC will recruit a cohort of 900 participants, distributed in approximately 20 Primary Care Centers (PCC) for each FC (with an average of 53 participants recruited per PCC). In each PCC, 5 to 10 Primary Care physicians are recruiting and following participants. Our experience with other studies is that 5 new patients can be recruited daily in each FC. Taking into account holiday periods and failures of invited participants to attend the scheduled visits, we estimate that each FC can recruit 90 new participants per month, with this inclusion rate the targeted sample size of 9,000 participants will be reached in June 2005.

D 2.3 Follow-up

Participants recruited in late 2003 will be followed for up to 5+ years and will have the longest follow-up. But participants entering the study after December 2004 will be followed only for less than 4 years, thus having the shortest follow-up. Consequently, we expect a median follow-up of 4 years.

|  | 2003 | 2004 | 2005 | 2006 | 2007 | 2008 | 2009 |
| --- | --- | --- | --- | --- | --- | --- | --- |
| Recruitment | Oct-------------------------Jun | | | | | | |
| Longest Follow-up | Oct---------------------------------------------------------------------------Dec (>5 yr) | | | | | | |
| Shortest Follow-up | Jun---------------------------------------------Dec (3.5 yr) | | | | | | |

D. 2.4 Analysis/closeout

Clinical closeout will take place by December 31, 2008. During January to March 2009, the ascertainment of outcomes, laboratory measurements, data entry, and respond-to-data edits will be completed, and data will be prepared for statistical analysis. Statistical analyses and writing of the reports for scientific publications will be done during the rest of the year 2009 and part of 2010.

D. 3 Study population / eligibility criteria

Trial participants will consist of 9,000 community-dwelling high-risk persons, with ages 55 to 80 years for men and 60 to 80 years for women. They should be free of cardiovascular disease and meet at least one of the two following criteria: a) or b) in D. 3 .1.

D. 3.1 Inclusion criteria: either a) or b) should be met.

a) Type 2 diabetes. Diagnosis of diabetes is based on at least one of the following criteria:

-Current treatment with insulin or oral hypoglycemic drugs.

-Fasting glucose > 126 mg/dl (fasting is defined as no caloric intake at least for 8 hours).

-Casual glucose > 200 mg/dl with polyuria, polydipsia, or unexplained weight loss.

-Glucose > 200 mg/dl in two measurements after an oral glucose tolerance test

OR

b) Three or more of the following risk factors:-current smoker (>1 cig/day during the last month)

-HBP (systolic BP>=140 or diastolic BP>=90 mmHg or under antihypertensive medication)

-LDL-cholesterol >= 160 mg/dl

-HDL-cholesterol <= 40 mg/dl independently of lipid-lowering therapy

-body mass index >=25 kg/m^2^

-family history of premature CHD (definite myocardial infarction or sudden death before 55 years in father or male 1^st^-degree relative, or before 65 years in mother or female 1^st^-degree relative)

-If the HDL-cholesterol level is >=60 mg/dL, one risk factor should be subtracted.

D. 3.2 Exclusion criteria. Major exclusion criteria are:

- Documented history of previous cardiovascular disease, including CHD (angina, myocardial infarction, coronary revascularization procedures or existence of abnormal Q waves in the electrocardiogram (EKG)), stroke (either ischemic or hemorrhagic, including transient ischemic attacks), and clinical peripheral artery disease with symptoms of intermittent claudication.
- Severe medical condition that may impair the ability of the person to participate in a nutrition intervention study (e.g. digestive disease with fat intolerance, advanced malignancy, or major neurological, psychiatric or endocrine disease)
- Any other medical condition thought to limit survival to less than 1 year.
- Immunodeficiency or HIV-positive status.
- Illegal drug use or chronic alcoholism or total daily alcohol intake >80 g/d.
- Body mass index > 40 kg/m^2^.
- Difficulties or major inconvenience to change dietary habits
- Impossibility to follow a Mediterranean-type diet, for religious reasons or due to the presence of disorders of chewing or swallowing (e.g., difficulties to consume nuts)
- A low predicted likelihood to change dietary habits according to the Prochaska and DiClemente stages of change model (Nigg, 1999).
- History of food allergy with hypersensitivity to any of the components of olive oil or nuts.
- Participation in any drug trial or use of any investigational drug within the last year.
- Institutionalized patients for chronic care, those who lack autonomy, are unable to walk, lack a stable address, or are unable to attend visits in the PCC every 3 months.
- Illiteracy.
- Patients with an acute infection or inflammation (e.g., pneumonia) are allowed to participate in the study 3 months after the resolution of their condition.

D. 4 Recruitment

Most FC have considerable experience and a successful track record of recruiting participants for both epidemiological studies and clinical trials. In addition, the participation of Primary Care Physicians will ensure a high enrollment rate. Because these physicians are responsible for the usual medical care of potential participants and they are aware of their risk factors, no potential ethical conflict regarding confidentiality exists at the stage of identification of suitable participants for the trial. This process starts with extracting their names from the PCC records. Most PCC (over 70 %) participating in the study have computer-based records of patients, making the selection relatively simple. Our experience during the pilot study indicates that the easiest way to select high-risk subjects is to obtain a complete database of persons with either diabetes or HBP fulfilling also the eligible gender-specific age ranges. When computer-based records are not available, Primary Care physicians develop a list of suitable candidates. The clinical records of these persons are then individually reviewed to exclude those who do not meet eligibility criteria. Candidates are contacted by telephone and are invited to attend a visit in the PCC, where the purpose and characteristics of the study are explained and an informed consent is requested (see below). Alternatively, the Primary Care physician may personally invite candidates to participate on the occasion of scheduled visits for any medical reason. A brief general explanation of the study, including the possibility that they may receive free allowances of olive oil or nuts for the duration of the trial is given in this first visit. Our experience has been that >70 % of candidates approached in this way agree to return for the screening visit.

D. 5 Participants’ visits

Participants’ eligibility for the trial is determined by review of their clinical record and by two formal screening visits. In these visits, questionnaires are filled in, a clinical history is taken, anthropometric and BP measurements are made, and fasting blood for biochemical analyses is obtained. Information collected in the screening visits will also provide baseline data for subsequent analyses of the effects of the interventions on intermediary biomarkers and risk factors.

- Pre-Screening Evaluation: After review of clinical records, eligible candidates are contacted by telephone to know if they are both capable and willing to participate in the study. Those giving a positive response are scheduled for the first visit. Data on participation proportion are collected.

- Screening visit 1: The visit, performed by the Primary Care physician, serves to identify inclusion/exclusion criteria in a more comprehensive manner. This 15-30 minute visit includes:

a) A face-to-face administration of a 26-item questionnaire to inquire about the medical conditions and risk factors related to eligibility, including assessment of the willingness to make diet changes (Prochaska model). b) A review of the last EKG if available in the clinical record. If no EKG has been performed within the last year, an EKG is done during this visit. c) If the candidate meets all the requirements (including EKG data), an informed consent form is given to him/her for signing after detailed explanation of all procedures and of the anticipated time commitment. The informed consent comprises two parts, one for trial participation and biochemical analyses and another for DNA collection for genetic analyses of stored samples. d) The following forms and questionnaires are provided to be read and completed at home:

-a detailed written explanation of the study

-a food frequency questionnaire (FFQ) with 137 items plus vitamin/minerals supplements (adapted from the Willett questionnaire and validated in Spain, see Martin-Moreno, 1993), in addition to specific questions about patterns of alcohol consumption.

-The Minnesota physical activity questionnaire (validated Spanish version, Elosua, 1994 & 2000).

e) The participant is instructed to collect toe-nail specimens and bring them in the next visit.

f) The next visit (screening visit 2) is scheduled and the candidate is told to attend it after an overnight fast for blood extraction.

After the first screening visit, the participant is randomized to one of three diet groups.

- Randomization: The study nurse randomly assigns each participant to the corresponding intervention group following tables of random allocation according to the recruitment order in blocks of 50 participants, balanced by sex and age group (< 70 years and > 70 years). These tables have been centrally elaborated by the Coordinating Unit and provide a stratified random sequence of allocation for each FC using closed envelopes. The four strata for stratified randomization are built according to gender and age (cut-off point: 70 years). The Primary Care physicians do not participate in the process of randomization. The study nurses are independent of the nurse staff of the PCC. Therefore, they are not involved in the usual clinical care of participants, their first and exclusive role being to collect the data for the PREDIMED trial.

- Screening visit 2: This 1 hour visit includes the following:

a) A simplified assessment of adherence to the MeDiet (14-item questionnaire). b) In a face-to-face interview with the candidate, the dietitian explains again in detail the purpose and development of the study. c) The dietitian reviews (and completes with the candidate if needed) the FFQ and physical activity questionnaires. Alternatively, she helps the candidate who had difficulties at home to fill in the questionnaires during the visit. d) The nurse measures the weight, height, waist circumference, and BP, and performs a measurement of the ankle-brachial blood pressure index. e) The nurse performs a venipuncture, obtains and handles blood samples, and proceeds to prepare the specified serum, plasma, and buffy-coat aliquots. f) A urine sample and toe nail specimens are collected by the nurse. g) A 47-item general questionnaire collecting information about current medication and risk factors is filled-in. Information to fill this questionnaire is also abstracted from clinical records by the research nurse.

D.6 Intervention

The PREDIMED dietitians are directly responsible for the dietary intervention. All PREDIMED dietitians are registered dietitians, trained and certified to deliver the PREDIMED intervention protocol. Before the implementation of the protocol, training consisted of approximately 24 hours of initial theoretical and practical group discussion with experts in nutrition education and discussion in between 3 to 5 conference calls to review and improve the protocol. These calls are continued bimonthly throughout the study. During the calls each dietitian discusses her practice sessions with the team, and together the group identifies problems and solutions in protocol implementation. Feedback and discussion also occur among the dietitians and the center coordinators, and between center coordinators and the Data Manager, especially after data from FFQ and objective biochemical measurements (in a random sample of 10% participants) of compliance are analyzed.

D. 6. 1 Control diet group: After screening visit 2, participants randomized to the Control diet group have an interview with a PREDIMED dietitian. This interview includes:

a) A simplified assessment of adherence to the MeDiet (14-item questionnaire). b) Brief personal recommendations to follow a low-fat diet (AHA guidelines). For total fat intake these recommendations are in some way opposite to those given to the participants in the 2 MeDiet intervention groups). c) A leaflet with written recommendations to follow a low-fat diet is given). No further visits are scheduled for the participants allocated to this group until the 1-year follow-up evaluation.

D. 6. 2 Intervention groups

D. 6.2.1 Individual motivational interview with a PREDIMED dietitian

After screening visit 2, participants randomized to either of the two MeDiet intervention groups have a face-to-face interview with the dietitian. This interview includes:

1. The 14-item questionnaire of adherence to the MeDiet (See Appendix).
2. Personal individual recommendations for changes to be introduced in the participant’s diet in order to achieve a personalized goal. The dietitian provides a comprehensive number of reasons to adopt a MeDiet, highlighting the advantages of following this diet, rather than the risks of not adhering to it, and transmitting a positive message with stress on the particular benefits for diabetic patients and for those at high cardiovascular risk. Our previous experience with diabetic patients using this approach for a behavioral intervention to quit smoking in Primary Care has been successful (Canga, 2000). We also have experience in using the stages of change model for dietary change (Lopez-Azpiazu, 2000). The dietitian personalizes the message by adapting it to the patient’s clinical condition, preferences, and beliefs. The training of the PREDIMED dietitians emphasized the holistic approach to lifestyle change in order to tailor the intervention to nutritional assessment and individual needs, encourage adherence to the MeDiet, transmit a sense of empowerment, and, importantly, feel a self-reward for each upward step in the 14-point MedDiet score. A contracting procedure is used and a negotiated change in diet is the targeted goal, working with the subject to determine what he or she considers an attainable goal. The focus can be shifted from changing portion sizes to changing frequency of intake or to changes in cooking methods. Accomplishments in the previous months, even if minor, are considered as support to provide further empowerment and self-reward. The usefulness and effectiveness of this approach has been shown in an even larger randomized trial currently conducted in the US that is aimed instead to reduce fat intake (Mossavar-Rahmani, 2004; Patterson, 2003). Importantly, caution is taken to make sure that participants with diabetes, overweight/obesity, and/or hyperlipidemia may not receive contradictory dietary advice from other health professionals external to the PREDIMED trial. The PCC physicians, who are responsible for the health care of participants, are aware of this caution. Because unsaturated fats like those contained in olive oil and nuts are still perceived as fattening by some nutrition experts, it is particularly important to allay the fear of an eventual weight gain that might have both the person who is on a weight-management program and his/her nutritionist. This is done by tactful exposition of recent scientific evidences (McManus, 2001; Ros, 2003; Sabaté, 2003; Garcia-Lorda, 2003) and, on from June 2004, by explaining that body weight did not change after 3 months of MeDiet intervention in the pilot phase of the PREDIMED study.
3. A leaflet with written information about the main food components and cooking habits of the MeDiet, together with recommendations on the desired frequency of intake of specific foods, is given to the each participant. Participants assigned to the MeDiet group receive an additional leaflet on health benefits, use, and conservation of olive oil, while those in the MeDiet+Nuts are given a leaflet with similar information regarding nuts, with emphasis on the three nut types used in the trial (walnuts, hazelnuts, and almonds).

d) The participant is scheduled for a group session in the next 1-2 weeks. The visit ends with an agreement to participate in the group session.

D. 6.2.2. Group sessions. The PREDIMED dietitian runs the sessions. No more than 20 participants may attend. Separated sessions are organized for each intervention group (MeDiet or MeDiet+Nuts). The group session includes:

a) An introductory talk by the dietitian to remind the 14-item score.

b) Clarification of possible doubts about face-to-face counseling or written material.

c) The following written material is given to each participant and discussed with them:

-Description of 4-5 food items typical of the MeDiet and adapted to the season of the year.

-A quantitative 1-week buying list of food items, according to the season of the year.

-A weekly plan of meals (with detailed menus) adapted to the buying list.

-The cooking recipes for cuisine practices according to the suggested menus.

d) The needed amount of either olive oil (15 liters = 1 liter/week for 15 weeks) or sachets of walnuts, hazelnuts, and almonds (1,350 g walnuts = 15 g/d; 675 g hazelnuts = 7.5 g/d, and 675 g almonds = 7.5 g/d, allotment for 90 days) are provided to participants, together with instructions about their use and conservation. To improve compliance, a 500-g pack of walnuts is given to each family unit.

e) The dietitian facilitates the exchange of messages and ideas included in section D. 6.2.1 b) among the members of the group.

f) The contact ends with an agreement to participate in the next visit (in the next 3 months).

In the MeDiet+Nuts group we offer participants three kinds of nuts: walnuts, hazelnuts, and almonds, instead of providing only one type of nut, because we have received funding from the nut industry to provide the three of them. As stronger evidence may support that ALA-rich walnuts can offer special advantages in cardiovascular prevention, we are supplying a higher amount of walnuts.

D. 6.2.3. Follow-up visits and reiteration of individual and group sessions

The individual and group visits are repeated every 3 months with the same contents (D.6.2.1 and D.6.2.2). Each visit includes three steps: assessment, intervention, and future directions.

D. 7 Description of intervention diets

Our main focus is in changing the dietary pattern instead of focusing in changes in macronutrients. Total fat intake for the 2 intervention groups is *ad libitum* (a high fat intake is allowed, as long as most fat is derived from fatty fish and vegetable sources, particularly olive oil or nuts). There is also no specific energy restriction. The two intervention diets will strongly differ from the diet recommended to the Control group in relevant micronutrients, mainly ALA present in walnuts and polyphenols in EVOO, together with flavonoids, other polyphenols, and sterols present in nuts and EVOO.

The common general guidelines to follow the MeDiet that PREDIMED dietitians provide to participants in the two intervention groups include the following positive recommendations: a) abundant use of olive oil for cooking and dressing dishes; b) consumption of >=2 daily servings of vegetables (at least one of them as fresh vegetables in a salad), without counting garnishing c) >=2-3 daily serving of fresh fruits (including natural juices); d) >=3 weekly servings of legumes; e) >=3 weekly servings of fish or seafood (at least one serving of fatty fish); f) >=1 weekly serving of nuts or seeds; g) select white meats (poultry without skin or rabbit) instead of red meats or processed meats (burgers, sausages); h) cook regularly (at least twice a week) with tomato, garlic and onion adding or not other aromatic herbs, and dress vegetables, pasta, rice and other dishes with tomato, garlic and onion adding or not other aromatic herbs. This sauce is made by slowly simmering the minced ingredients with abundant olive oil. Negative recommendations are also given to eliminate or drastically limit the consumption of the following foods: cream, butter, margarine, cold meat, paté, duck, carbonated and/ or sugared beverages, pastries, industrial bakery products (such as cakes, donuts or cookies), industrial desserts (puddings, custard), French fries or potato chips, and out-of-home pre cooked cakes and sweets.

The dietitians insist that 2 main meals per day should be eaten (seated at a table, lasting more than 20 minutes); for usual drinkers, the main source of alcohol should be wine (maximum 300 ml, 1-3 glasses of wine per day). If wine intake is usual, a recommendation to drink a glass of wine per day (bigger for men, 150 cc, than for women, 100 cc) during meals is given. *Ad libitum* consumption is allowed for the following food items: nuts (raw and unsalted), eggs, fish (recommended for daily intake), seafood, low fat cheese and whole-grain cereals. Limited consumption (=<1 serving per week) is advised for cured ham, red meat (after removing all the visible fat), chocolate (only black chocolate, with more than 50% cocoa), cured or fatty types of cheese.

D. 8.1 Menu development

Most of the studies that have examined the Mediterranean diet have been conducted under relatively controlled conditions, with most foods and dishes given to a reduced sample of participants by the research team. The PREDIMED trial represents a further step to obtain more relevant information for public health use, because the nutritional intervention is undertaken in free-living persons, who receive information, motivation, support and empowerment to modify their food habits in a real-life context, i.e. they continue to buy their foods and cook their meals. Such an intervention provides a realistic scenario that may be easily applied to public health policies. However, since palatability of meals is extremely important to ensure compliance, menus and recipes with these characteristics for the two intervention diets have been developed. Menus are designed to meet the nutrient targets. They are provided to the participants and they may learn to prepare the menus using the recipes and the information given by the dietitian.

D. 8.2. Food supply and distribution

A 15-liter supply of EVOO rich in polyphenols (®Hojiblanca, Spain) is provided every 3 months to each participant in the MeDiet group. Similarly, every 3 months a supply of 1,350-g walnuts (®California Walnut Commission, Sacramento, Cal), 675-g almonds (®Borges SA, Reus, Spain), and 675 h hazelnuts (®La Morella Nuts, Reus, Spain) is provided to each participant assigned to the MeDiet+Nuts group. Depending on personal preference and convenience, the participants collect the free foods at the PCC at the time of the 3-monthly group session or have the supplies shipped to their homes. The three nut industry companies are committed to supply for free the nuts used in the study until its termination. The Hojiblanca Company has agreed to supply the olive oil for free during the year 2004, and our agreement with the company has to be renewed every year. We also have approached the International Olive Oil Council as a second option for additional supply of olive oil. None of the investigators has any conflict of interest with these food companies, and there is a complete freedom to publish the findings of the study.

D. 8.3. Adherence promotion

Efforts to promote adherence began at the earliest stages of the study. During screening and orientation, participants are repeatedly provided with information about key features of the study and with the concept of MeDiets. At the first screening visit, the attitude towards dietary change is assessed in the eligibility questionnaire. Individuals must be willing to change their diets, otherwise they are excluded. The dietitian-led motivational and education intervention includes both individual and group sessions every 3 months, totaling more than 32 intervention visits during the trial. Additional written material is provided. Furthermore, the free distribution and supply of key food items ensures a high adherence to the intended diets during the trial. Acceptance of the intervention protocol is increased because no specific caloric restriction is imposed and participants are allowed an *ad libitum* fat intake, if it comes from olive oil, nuts, other plant-sources or fatty fish. However, after randomization, every effort is made to promote adherence. In many instances, these efforts are tailored to the specific needs of the participant (e.g. food items delivered to home or work).

D. 8.4. Compliance assessment

The yearly administered FFQ will provide information about compliance and attainment by participants of the nutrients targets. Although the FFQ that we are using has been previously validated in Spain (Martin-Moreno, 1993), we will conduct a sub-study during 2004-05 to validate and calibrate it again. In this substudy we will include 150 additional participants, not participating in the major trial, but recruited in the same way and with a similar age and gender distribution, who will complete 2 FFQ, one at baseline and another at the end of 1 year, together with four 3-day food records separated by 3 months. This process will ensure a better quality in the measurement of actual diets and will allow corrections for measurement errors in the FFQ.

At any rate, the information extracted from the FFQ will only provide a subjective assessment of compliance. To obtain also an objective evaluation, we will measure biological markers of compliance in a random subset of participants from the three arms of trial. In a random sample of 10% of participants, a blood sample and urine aliquots will be used to blindly ascertain the following markers of compliance: a) plasma fatty acid composition (specially oleic and ALA, which are reliable indicators of MUFA and walnut consumption, respectively); b) urinary tyrosol and hydroxytyrosol (EVOO); c) urinary resveratrol and ethanol (wine and other alcoholic beverages). To relate these measurements to the time of intake, participants are asked the time spent since they last consumed the specific foods when blood and urine samples are taken.

D. 8 Measurements

Table 1 displays major measurements and data collection activities by visit.

Table 1. Overview of measurement scheduled in the PREDIMED trial.

|  | BASELINE | YEAR-1 | YEAR-2 | YEAR-3 | YEAR-4 |
| --- | --- | --- | --- | --- | --- |
| 1. ELIGIBILITY QUESTIONNAIRE | X |  |  |  |  |
| 2. GENERAL QUESTIONNAIRE* | X |  |  |  |  |
| 3. FOOD FREQ. QUESTIONNAIRE | X | X | X | X | X |
| 4. PHYS. ACTIV. QUESTIONNAIRE | X | X | X | X | X |
| 5. 14-ITEMS SCORE OF MEDIET | X | X | X | X | X |
| 6. FOLLOW-UP QUESTIONNAIRE* |  | X | X | X | X |
| 7. EKG | X | X | X | X | X |
| 8. BLOOD SAMPLE | X | X | X | X | X |
| 9. URINE SAMPLE | X | X | X | X | X |
| 10. TOE NAIL SAMPLE | X |  |  |  |  |

* Includes measurements of weight, height, waist circumference, BP, and ankle-brachial blood pressure index.

D. 8. 1 Questionnaires

Five questionnaires (#1 to 5 in table 1) have already been described in this proposal. The PREDIMED dietitians are responsible for the accurate filling of the questionnaires.

The follow-up questionnaire (#6 in table 1) collects information about the following issues:

-Socio-demographic variables (changes since baseline): 7 items.

-Changes in smoking habits: 3 items.

-New medical diagnoses of diabetes, hyperlipidemia or hypertension: 3 items.

-New medical diagnoses of cardiovascular events: 10 items.

-Inquiries about non-cardiovascular complications of diabetes: 3 items.

-Other medical conditions: 3 items.

-Current use of medication (including doses): 20 items.

-Time since the last intake of EVOO.

-Time since the last intake of wine or other alcoholic beverages.

D. 8. 2 Blood pressure (BP) and anthropometric measurements

BP and body weight and height are measured by PREDIMED nurses, who are trained and certified for these measurements. We use numerous quality control procedures to promote accurate measurements. We are tracking the performance of nurses in collecting this information (e.g. checking for digit preference and excessive variation on replication measurements). We are training, certifying and recertifying nurses every 6 months. For BP measurement, participants rest quietly for five minutes in the seated position. A validated semi-automatic sphygmomanometer (Omron HEM-705CP) is used for the PREDIMED trial. An appropriate sized cuff is applied after measurement of arm circumference. A pulse obliteration pressure is obtained. At each visit, 3 measurements will be obtained, separated by 2 minutes. The average of second and third measurement is written in the data collection form. If both measurements differ more than 5 mmHg, the whole procedure is repeated and additional BP readings are averaged. The systolic blood pressure (by Doppler probe) is taken in brachial, posterior tibial, and dorsalis pedis arteries to compute the ankle-brachial blood pressure index. Weight is measured using a calibrated balance beam scale with the subject barefoot and wearing light clothes. Height is measured by the nurse using a wall-mounted calibrated stadiometer. Waist circumference is measured using an anthropometric measuring tape, at a horizontal plane midway between the lowest rib and the iliac crest.

D. 8. 3 Electrocardiograms (EKG)

At each yearly visit, the nurse obtains from the clinical record the last available EKG, and collects 2 copies. If no EKG has been taken during the last year, she communicates with the PCC physician to perform a new EKG and 2 copies are included in the PREDIMED file of the participant.

D. 8. 4 Extraction, processing and storage of biological samples

The PREDIMED nurses are directly responsible for collection, processing and storage of biological specimens. All PREDIMED nurses are experienced and registered nurses trained and certified to perform the specimen collection protocol. Training, before starting the trial, consisted of approximately 4 hours of theoretical information and 4 hours of practical instruction. Blood samples are collected at baseline and in yearly follow-up visits according to the protocol depicted in table 2.

Table 2. Blood samples taken each year.

|  | Number of tubes | | Volume (ml) | |  | |
| --- | --- | --- | --- | --- | --- | --- |
| Glass tube K3E EDTA | | 4 | 4.5 | EDTA Plasma + buffy coat | |  |
| Plastic tube K3E EDTA (cold) | | 1 | 3 | Cold EDTA plasma (pre-refrigerated tube) | |  |
| Glass tube 9NC Citrate | | 1 | 4.5 | Citrate Plasma + buffy coat | |  |
| Gel-Glass tube SST | | 2 | 4 | Serum (one of them light-protected for Hcy) | |  |
| Glass tube K3E EDTA* | | 1 | 5 |  |  | |
| TOTAL | | 9 | 38.5 |  | |

*Only a random sample (10%) is analyzed for biomarkers of compliance: the remaining is stored for future analyses.

The plastic tube K3E EDTA (cold) and 1 gel-glass tube SST (for homocysteine) will be refrigerated in ice prior to blood collection; after blood is collected, the tubes will be kept cold in the ice container. Serum, citrate plasma and EDTA plasma samples will be distributed in aliquots of 650 microliters and stored at -80°C for later analyses in the central laboratory. Biochemical measurements will be performed blindly and in the same batch for consecutive samples of each participant. Every FC has acquired a refrigerator with enough capacity to store these specimens. A urine sample will be taken yearly from each participant, and 16 aliquots (500 microliters) will be stored at -80ºC. Depending on the available funding for the PREDIMED trial, some of the blood samples could be omitted on years 1-4. All biological samples will be processed at each FC not later than 2 hours after collection. During transport from the PCC to the FC laboratory, they will be stored in a portable cooler (-4ºC). A clip of each toenail of each participant is stored at room temperature.

In addition, a complete blood count and routine biochemical measurements will be performed yearly in the PCC (fasting blood glucose, uric acid, ALT, AST, gamma-glutamyl transpeptidase, alkaline phosphatases, bilirubin, creatinine, BUN; total, HDL-, and LDL-cholesterol; triglycerides, total protein, and albumin) together with a routine urine exam including the albumin/creatinine ratio in a recent sample. Serum biomarkers of inflammatory status (VCAM-1, ICAM-1 and IL-6) and oxidative stress (MDA) will be determined in a subsample of participants. In addition, in a random sample of participants, genomic DNA has been isolated from leucocytes and some polymorphism in candidate genes that may modulate the cardiovascular response to diet (hepatic lipase, lipoproteinlipase, PPARG, APOE, paraoxonase, MTHFR, and others) have been analyzed by PCR and further allelic discrimination. Some members of our team have played a relevant role in identifying gene-diet interactions which can be of interest in our project (Ordovas, 2001; Corella, 2002).

D. 8.5 Outcome ascertainment

Outcomes will be determined by review by the Clinical End Point Committee. This panel will be blinded to the intervention group. The primary outcome is a composite of major cardiovascular events that includes cardiovascular death, definite non-fatal myocardial infarction (MI), coronary revascularization procedures, and ischemic stroke. Cardiovascular deaths will be ascertained by the Clinical Event Subcommittee from clinical registers on the basis of the clinical record and a death certificate listing an International Classification of Diseases code corresponding to any cardiovascular death (CHD or stroke). A definite non-fatal MI refers to a report of a clinical MI by a clinical center that meets the criteria for MI described in the Manual of Operations. Myocardial infarction is defined by the presence of symptoms suggestive of ischemia or infarction, with either electrocardiographic evidence (new Q waves in two or more leads) or cardiac-marker evidence of infarction, according to the standard American College of Cardiology definition (Cannon, 2001).

-Revascularization procedures include coronary by-pass, angioplasty, and thrombolytic procedures.

-Stroke diagnosis is based on rapid onset of a neurological deficit lasting more than 24 hours, supported by imaging studies (CT or MR scans).

Secondary analyses will be done for death by any cause, heart failure with pulmonary edema, new-onset diabetes mellitus, dementia, and cancer incidence other than non-melanoma skin cancer. The Clinical End Point Committee will follow standard procedures for attribution of these outcomes, as specified in the Manual of Operations.

D. 9 Statistical Analysis

There will be two primary analyses. The first will compare the incidence of major cardiovascular events in the MeDiet group with the control diet group. The second analysis will compare the same outcome between the MeDiet+Nuts group and the control diet group. We do not expect to have enough statistical power for the comparison of one intervention (MeDiet) with the other (MeDiet+nuts). Therefore, the aforementioned two analyses will be the primary measures of efficacy. The implications of multiple comparison issues are minimal with this planned analysis. P-values will be always two-sided. Primary analyses will be based in the intention-to-treat principle. If the results are as expected (see estimates for RR in C.3.4), a useful potential application for public health will be obtained because several alternatives can be offered to the population. For subjects who do not follow a MeDiet, this pattern can be recommended. For subjects who already follow a MeDiet, adding nuts can be recommended. For those who do not feel able to follow a MeDiet, they can be recommended to supplement their diet with nuts. Secondary analyses will be done for death by any cause, heart failure with pulmonary edema, new-onset diabetes mellitus, new-onset dementia, and cancer incidence other than non-melanoma skin cancer. We will analyze also changes in blood lipids, BP, markers of inflammation and other intermediate markers of cardiovascular risk in a subsample of participants, selecting a random sample of at least 300 participants from each group. These comparisons will allow us to better understand the mechanisms by which the experimental diet acts on cardiovascular risk. Depending on the available funding at the closure of the trial we will expand the number of determinations and the number of subjects. Analysis of variance with Dunnet post-hoc contrasts versus the control group will be the primary comparison in these analyses. In addition, gene-diet interaction in determining intermediate and final phenotypes will be examined in a sub-sample of participants.

The Data and Knowledge Management Center (DKMC) will be responsible for preparing reports to monitor the progress of the study for the Steering Committee and the Data and Safety Monitoring Board (DSMB). The main variable for analysis will be time from randomization to event. For a given outcome, the time of the event will be defined as the number of days from randomization to the first post-randomization diagnosis, as determined by the adjudicator. For silent MI, the date of the follow-up EKG will be applied. Participants without a diagnosis will be censored at the time of last follow-up contact. The generally accepted method for analyzing this type of data is the logrank statistic. It has the advantage of requiring no assumptions other than the random assignment of the intervention. This analysis will be the primary measure of the success or failure of the trial. However, if baseline imbalances between groups are observed for any of the main CHD risk factors, a Cox proportional hazard analysis will be conducted, stratified by FC, age, gender and major risk factors. Primary outcome comparisons will be estimated as hazard ratios. Adjustment for other relevant variables (body mass index, physical activity, education level, marital status) will be also applied.

To assess the progress of the daily operation of the study, the DKMC will prepare routine reports for the Steering Committee. These reports will focus on the general status of a) participant recruitment; b) participant adherence; c) quality control; d) clinical outcome data at each center. No endpoint will be included in the Steering Committee Reports.

The DSMB reports will be prepared for each DSMB review and will be tailored to meet the needs of the Committee, including: a) general progress of study and recruitment (person-years of follow-up in comparison to targets stated in advance); b) endpoints (group comparisons with respect to both the primary outcome, total mortality and the other secondary outcomes); c) adherence (biochemical markers and FFQ data of compliance and comparison with targets for nutrients and groups of foods); d) data quality (digit preference, variability, outliers). In these sections, data will be provided for the study as a whole and, where appropriate, separately for each FC. Reports will be mailed to members of the DSMB two weeks prior to the meeting or to the date of review. Steps will be taken to insure security and confidentiality, including distribution by certified mail and enactment of a return policy of all reports. Comparison of groups with respect to major outcomes will be updated two days before the meeting so that the DSMB will have the most up-to-date data possible.

Data will be analyzed at the statistical unit of the DKMC with the support of the statistical units of IMIM (Barcelona) and University of Navarra (Pamplona), which will reassure the quality of the results by following well-established analytical quality assessment procedures. Subgroups specified prospectively for analysis of intervention effects are: a) gender; b) age; c) diabetes status at baseline; d) plasma LDL and HDL; e) hypertension; f) smoking; g) pre-trial level of adherence to MeDiets; h) center (FC). The data from the PREDIMED trial will be analyzed at 5 intervals (4 interim analyses and the final analysis). After interim analyses the study will be terminated or extended if warranted. There are 3 potential reasons for early ending of the trial: a) efficacy of MeDiet may be demonstrated; b) harmful effects of the intervention may be discovered; c) there may be no hope for a reasonable evaluation of the proposed hypothesis (i.e., if a small intervention differential exists, power may be seriously compromised and early termination of the trial may be considered). In the case of a greater than expected observed benefit, early termination should be considered only if the intervention effect is great, and a conservative rule to stop the trial has been adopted. We have selected the O'Brien-Fleming boundaries (O'Brien, 1979), using asymmetric lower and upper boundaries (see below). The 4 interim analyses to ascertain the continuation of the PREDIMED trial will be conducted after 1, 2, 3 and 3.5 years of median follow-up. The 2-sided p-values for stopping the trial at each interim analysis (1^st^ to 4^th^) are respectively 5*10^-6^, 0.001, 0.009 and 0.02 for benefit and 9*10^-5^, 0.005, 0.02 and 0.05 for adverse effects (each p value corresponding to the a normal distribution value z_i_=(P*(5/i))^0.5^, where i is the i^th^ comparison and P is taken from O'Brien, 1979 for alpha=0.05 and alpha=0.10). The actual recommendation regarding stopping or continuing the trial will be made by the DSMB. The statistical test used is important but it will be one of many considerations in making these decisions.

D.10 Sample size

A sample size of 9,000 participants will provide sufficient statistical power to investigate the effect of the MeDiet+EVOO and MeDiet+Nuts on cardiovascular events. The sample size estimates were computed by comparing two binomial proportions representing the event rates in one of the treatment groups and in the control group, using the following equation:

N = 2 (z_(1-beta)_+z_(1-alpha/2)_)^2^[P_1_ (1-P_1_)+ P_2_ (1-P_2_)]/( P_1_ - P_2_)^2^.

where P_1_ and P_2_ are the proportion of participants that have events after 4 years in the control and intervention groups, respectively. We assumed a 80% power (1-beta=0.8) and (1-alpha/2=0.975) for a 2-sided p-value of 0.05. To estimate the projected cumulative incidence in the control group, we used the Framingham tables that predict 10-year absolute risks for CHD in the next ten years. For a 69-year man and a 69-year woman with 3 risk factors (e.g. smoker, stage I hypertensive, total cholesterol=260 mg/dl) the 10-year risks are 21% and 15%, respectively: Averaging and adapting these figures to a 4-year follow-up, a 7% average CHD absolute risk can be assumed. The higher sensitivity in the definition of myocardial infarction (i.e., including troponin) may compensate for the lower risk that is expected in participants in a trial. Ischemic stroke is also included in the composite primary outcome, this adds a further 5% absolute risk, predicting a 12% overall event rate after 4 years in the control group. The minimum decrease in number of events in the intervention group that is desired to be detected is 20%. Then p_1_ = 0.12 and p_2_= (0.12)(0.8) = 0.096. The total required number of participants with these assumptions would be 2,625 in each group. We are including 3,000 subjects in each group to allow for 10% losses to follow-up (Table 3).

Table 3. Sample size considerations.

| Expected scenario at the end of trial | | | | | | | | | | | | | | | |
| --- | --- | --- | --- | --- | --- | --- | --- | --- | --- | --- | --- | --- | --- | --- | --- |
| Group | | | n recruited | | (90% follow-up)  n (followed-up) | | | | Expected  Events | | Minimum detectable RR (expected 95% CI) | | | Statistical  Power | |
| MeDiet | | | 3,000 | | 2,700 | | | | 302 | | 0.80 (0.69-0.92) | | | 86.7% | |
| MeDiet+Nuts | | | 3,000 | | 2,700 | | | | 302 | | 0.80 (0.69-0.92) | | | 86.7% | |
| Control group | | | 3,000 | | 2,700 | | | | 378 | | 1 (ref.) | | |  | |
| Power calculations: sensitivity analysis (AR=absolute risk in control group) | | | | | | | | | | | | | | | |
| 95% follow-up | | | | | | 90% follow-up | | | | | | 85% follow-up | | | |
| AR= | (0.14) | (0.12) | | (0.1) | | | (0.14) | (0.12) | | (0.1) | | (0.14) | (0.12) | | (0.1) |
| RR= 0.75 | 99% | 98% | | 95% | | | 99% | 97% | | 94% | | 98% | 97% | | 93% |
| RR= 0.80 | 93% | 88% | | 81% | | | 92% | 87% | | 79% | | 90% | 85% | | 77% |

RR= relative risk. CI= confidence interval. AR=absolute risk (control group).

In the sensitivity analysis for power calculations (Table 3) we present several assumptions regarding follow-up rate (95%, 90% and 85%), absolute risk after 4-year follow-up in the control group (0.14, 0.12 and 0.1) and relative risks (0.75, 0.80). For relative risks around 0.8 we will have enough power in the different potential scenarios. We believe that our assumptions are realistic, because previous trials and observational studies of MeDiets have found a stronger reduction in risk than what we are assuming here. The only arguable assumption might be an artificially high absolute risk for a Mediterranean population. However, the incidence of stroke in the Spanish population of these ages is substantial, and almost approximates the incidence of CHD. Unfortunately, there are no population registries to document the incidence of non-fatal cardiovascular disease, but according to the last available mortality data from Spain, CHD caused 38,688 deaths and stroke caused 36,420 deaths in year 2000 (http://cne.isciii.es). Therefore including stroke in the composite outcome of cardiovascular disease may almost double the expected number of events, estimated with the Framingham table of CHD risk. Furthermore, the use of survival analysis methods (log-rank test) will improve the statistical efficiency of the analyses and will substantially increase the power.

D. 11 Organizational structure

The project will be supervised by the DSMB. The Project Leader, Dr. Ramon Estruch (Hospital Clinico, Barcelona), is assisted by the Steering Committee to take the strategic decisions along the project life. The coordination, research and internal supervision activities of the project will be performed by the following three categories of units: coordination units, research and operative units, and subcommittees. Table 4 shows data of each unit. In summary, the research team includes 11 Field Centers (FC), two Data Management and Statistical Units (DMSU); a Data and Knowledge Management Center (DKMC); and 5 specialized laboratory units (SLU). The Steering Committee, chaired by Dr. Estruch, includes the coordinators of each of the 11 FC, the investigator responsible of the each SLU and the Data Manager. There are also the following organizational structures: a Diet Subcommittee, co-chaired by Dr. Martinez-Gonzalez (Navarra) and Dr. Ros (Barcelona), a Measurement Subcommittee, chaired by Dr. Covas IMIM-Barcelona), a Recruitment Subcommittee (chaired by Dr. Salas), a Clinical End Point Committee, chaired by Dr. Aros (Cardiologist, Txagorritxu Hospital, Vitoria) and will include a clinician from each FC. The Data and Safety Monitoring Board (DSMB) has been established and it includes the following external advisors: Dr. Valentin Fuster, Mont Sinai School of Medicine, New York; Dr. F. Xavier Pi-Sunyer, Columbia University College of Physicians and Surgeons, New York; Dr. Joan Sabaté, Loma Linda University, CA; Dr. Carlos A. González, Principal Investigator of the EPIC cohort in Barcelona, Spain. They will be convened to review the implementation of the protocol and to monitor trial progress on an annual basis. In addition, as stated, a mailed report will be sent them periodically with the pertinent analyses to ascertain the continuation of the PREDIMED trial. Most of the Committees will convene by conference calls and at in-person meetings (twice per year during years 1-4, once during year 5). The frequency of contacts will vary during the trial.

Table 4. Description of Project Organic Units.

| Categ. | Unit name | Responsibilities |
| --- | --- | --- |
| Coordina-tion | Administration | General management, accounting, task schedule management. |
|  | Technological Support | Technical and technological assistance in computing services, instrument installation and maintenance. |
|  | Data and Knowledge Mana-gement Center (DKMC) | Statistical analysis, data management, report elaboration, database management, software support tools development, security of information and computer systems. |
|  | Medical Support | Medical assistance in clinical tasks |
| Research and Operative | Barcelona University (Hospital Clinico) | FIELD CENTER (FC)-1: Patient recruitment, dietary behavioral intervention  Specialized Laboratory Unit (SLU)-1 |
|  | Barcelona, IMIM, Primary Health Care | FC-2: Patient recruitment, dietary behavioral intervention  Data Management and Statistical Unit (DMSU)-1, SLU-2 |
|  | Navarra University | FC-3 Patient recruitment, dietary behavioral intervention, DMSU-2, FFQ processing |
|  | Valencia University | FC-4: Patient recruitment, dietary behavioral intervention, genetic analysis, SLU-3 |
|  | Malaga University | FC-5: Patient recruitment, dietary behavioral intervention, laboratory work |
|  | Seville University | FC-6: Patient recruitment, dietary behavioral intervention |
|  | Seville Primary Health Care | FC-7: Patient recruitment, dietary behavioral intervention |
|  | Seville-CSIC* | SLU-4 |
|  | Mallorca, I. Balears University | FC-8:Patient recruitment, dietary behavioral intervention |
|  | Tarragona, Rovira Virgili Univ. | FC-9: Patient recruitment, dietary behavioral intervention, laboratory work |
|  | Vitoria, Txagorritxu Hospital | FC-10: Patient recruitment, dietary behavioral intervention |
|  | Madrid, Hospital Carlos III | FC-11: Patient recruitment, dietary behavioral intervention |
|  | Zaragoza University | SLU-5 |

*CSIC: Superior Council for Scientific Research. IMIM: Municipal Institute for Medical Research.

D. 12 Data management

The DKMC will be co-directed by the Data Manager, Dr. Covas, and Dr. Martinez-Gonzalez. With the support of the DMSUs (IMIM and Navarra University), they will develop and maintain the data base; and will provide statistical and trial monitoring support throughout the field work. Data from screening, intervention and follow-up visits will be entered on specific forms at the FC, and sent monthly to the Data Manager in the DKMC, who will send reports of missing or inappropriate entries, for clarification and resolution, to the FC coordinators every month. The Data Manager will also provide monthly reports to Dr. Estruch on the quality and completeness of the data, organized by type of visit (screening visit 1, 1-year follow-up visit, etc.) and by specific data form. At the end of each yearly visit in each FC, the Data Manager will verify the completeness of data for each individual.

A web based system of data access has been created (www.predimed.uji.es/drupal) where all the forms and datasets and published papers can be downloaded by authorized investigators. For privacy and security, an ID and password are required to access the data and the forms. This web-based system can be also used to send data to the Data Manager. Quality control reports will be generated for key aspects of the trial, e.g., digit preference and variability. This web-based system can be also used to send data to the Data Manager. Quality control reports will be generated for key aspects of the trial, e.g., digit preference and variability. To reduce data entry expenses and speed processing, the questionnaires and data forms can be optically scanned. The data forms will be entered in duplicate and missing data checks will be performed. After data entry, cross-form edit checks will also be performed. Data inconsistencies will be checked. Audits will be rerun periodically to detect unresolved problems. Standardized edit reports that summarize problems in the database provide an additional method of assuring data quality. To minimize the potential for error, we have developed a detailed Manual of Operations. In addition we will conduct annual training meetings for staff. The DKMC will monitor the performance of each FC and will recommend new or corrective procedures in case deficiencies are noted. Until the end of the trial, all FC will be masked to trial outcome data, with the exception of the statisticians, the Data Manager and the external DSMB. Due to the nature of the intervention, however, dietitians and nurses at each FC need to be unmasked to diet assignment. The Clinical Event Subcommittee will be blinded to participant allocation.

D. 13. Design considerations

In our deliberations, we considered alternative designs. One set of considerations related to the type of study (behavioral intervention versus feeding study). Controlled feeding studies are the best procedure to assess the biological effect of nutrients on intermediary markers of cardiovascular risk. However, the existence of multiple pathways for the effects of dietary exposures on CHD, some of them newly identified and still some of them that will be very likely identified in the next years may render intermediate surrogate markers of CHD risk as misleading. These considerations strengthen the need to study clinical outcomes. If clinical outcomes are to be observed, a controlled feeding design is completely unfeasible. Second, from a public health perspective, a behavioral intervention coupled with an easy (free) access to the supposedly healthy food represents a more realistic test of the effectiveness to be attained with public policies and health promotion activities in nutrition and public health. The purported benefits of diets rich in ALA led us to adopt the three group design, because we would assess the effect of two interventions, one with MeDiet and another with an ALA-rich MeDiet (walnuts are rich in ALA), both compared with a control group. We acknowledge this design has problems of feasibility. These problems would substantially increase should we adopt a factorial (2x2) design. Therefore we opted for a simpler, three-group randomization.

D.14 Limitations of the study

The control group is not receiving a special behavioral intervention and controls continued in fact following a diet rich in fat and not very different in its macronutrient composition from the diet of the intervention groups (although a sizeable amount of MUFA was derived from meat instead of olive oil or nuts). Therefore, the macronutrient composition of the compared diets did not provide a strong contrast. We acknowledge this limitation because controls belong to a Mediterranean culture and their food habits are not very far from the MeDiet. But we think that it would be unethical to strongly advice controls to get apart from this supposedly healthy diet. Instead, we are requesting additional funding to increase differences by using a comprehensive and expanded intervention in both experimental groups to attain further changes in their diet, thus maximizing the contrast.

We also acknowledge that changes in blood lipids and BP, although significant, were not very impressive. However, these changes are likely to increase with repeated dietary advice. Furthermore, the joint effect of minor changes in classical risk factors with dramatic changes in markers of inflammation can be associated with a substantial reduction in risk.

The main differential point in our design is that we are evaluating two MeDiets which are higher in fat than all diets assessed by previous trials, but in both of them the sources of fat are mainly MUFA. In addition, the effect of diets rich in polyphenols and phytosterols is also assessed. The rigorous methodology of the PREDIMED trial should convincingly answer what is certainly one of the major sources of controversy and unresolved issues regarding diet and cardiovascular risk, namely, the impact of replacing saturated fat with MUFA or with carbohydrate.

References

| Albert CM, et al (2002). Arch Intern Med 162:1382-7.  Anderson KJ, et al (2001). J Nutr 131:2837-42.  Ascherio A, et al. BMJ 313:84-90.  Baylin A, et al. (2003). Circulation 107:1586-91.  Bazzano LA, et al (2001). Arch Intern Med 161:2573-8.  Becker, 2002. Eur J Clin Invest 32:1-8.  Bray GA, Popkin BM (1998). Am J Clin Nutr 68:1157–73.  Canga N, et al (2000). Diabetes Care 23:1455-60  Cannon CP, et al (2001). J Am Coll Cardiol 38:2114-30.  Carluccio MA, et al (1999). Arterioscler Thromb Vasc Biol 19::220-8.  Carluccio MA, et al (2003). Arterioscler Thromb Vasc Biol 23:622-9.  Connor WE, Connor SL (1997). N Engl J Med 337:562-3.  Corella D, et al (2002). J Lipid Res 43:416-27.  Costacou T, et al (2003). Eur J Clin Nutr 57:1378-85.  De la Puerta, et al (1999). Biochem Pharmacol 57:445–9.  De la Puerta, et al (2001). Life Sci 69;1213–22.  De Lorgeril M, et al (1999). Circulation 99:779-85.  Desideri , et al (2002). Clin Chim Acta 320:5-9.  Djousse L, et al (2003). Am J Clin Nutr 77:819-25.  Ellsworth JL, et al (2001). Nutr Metab Cardiovasc Dis 11:372-7.  Elosua R, et al (2000). Med Sci Sports Exerc 32:1431-7.  Elosua R, et al (1994). Am J Epidemiol 139:1197-209.  Feldman EB (2002). J Nutr 132:1062S–1101S.  Ferrara LA, et al (2000). Arch Intern Med 160:837-42.  Ferro-Luzzi A, et al (2002). Eur J Clin Nutr 56:796-809.  Fraser GE, et al (1992). Arch Intern Med 152:1416-24.  Fuentes F, et al (2001). Ann Intern Med 134:1115-9.  Garcia-Lorda P, et al (2003). Eur J Clin Nutr 57:S8-11.  Guallar E, et al (1999). Arterioscler Thromb Vasc Biol 19:1111-8.  Haim , et al (2002). J Am Coll Cardiol 39:1133-8.  Hu FB, et al (1998). BMJ 317:1341-5.  Hu FB, et al (1999). Am J Clin Nutr 69:890-7.  Hu FB (2003). N Engl J Med 348:2595-6.  Hwang et al (1997). Circulation 96:4219-25.  Jacobs DR Jr, Steffen LM (2003). Am J Clin Nutr 78(Suppl):508-13S.  Jacques PF, Tucker KL (2001). Am J Clin Nutr 73:1-2.  Jequier E, Bray GA (2002). Am J Med 113(9B):41S–46S.  Joint WHO/FAO Expert Consultation on Diet, Nutrition and the Prevention of Chronic Diseases (2002: Geneva, Switzerland). WHO technical report series; 916. Geneva: WHO, 2003.  Kouris-Blazos A, et al (1999). Br J Nutr 82:57-61.  Kris-Etherton PM, et al (2001a). Circulation 103:1823-5.  Kris-Etherton PM, et al. (2001b). Nutr Rev 59:103-11.  Lasheras C, et al (2000). Am J Clin Nutr 71:987-92.  Lercker G, Rodriguez MT (2000). J Chromatogr A 881:105-29.  Lopez-Azpiazu I, et al (2000). Public Health 114;183-9.  Luc, et al (2003). Atherosclerosis 170:169-76.Malik, et al (2001). Lancet 358:971-6.  Manna C, et al (2002). J Agric Food Chem 50:6521-6.  Mangiapane EH, et al (1999). Br J Nutr 82:401-9.  Martinez-Gonzalez MA, et al (2002). Eur J Nutr 41:153-60. | Martinez-Gonzalez MA, Estruch R (2004a). Eur J Cancer Prev (in press).  Martin-Moreno JM, et al (1993). Int J Epidemiol 22: 512-9.  Marrugat J, et al (2004). Eur J Nutr 43:140-7.  Mata P, et al (1997). Arterioscler Thromb Vasc Biol 1::2088-95.  Mataix J, et al (1998). Free Radic Biol Med 24: 511-21.  McManus K, et al (2001). Int J Obes 25:1503-11.  Miles, et al (2001). Clin Sci (Lond) 100:91-100.  Mossavar-Rahmani Y, et al (2004). J Am Diet Assoc 104:76-85.  Nigg CR, et al (1999). Gerontologist 39:473-82.  O'Brien PC, Fleming RT (1979). Biometrics 35:549-56.  Oomen CM, et al (2001). Am J Clin Nutr 74:457-63.  Ordovas JM, et al (2002). Circulation 106:2315-21.  Panagiotakos DB, et al (2002). Prev Med 35:548-56.  Patterson RE, et al (2003). J Am Diet Assoc 103:454-60.  Perez-Jimenez F, et al (2002). Atherosclerosis 163:385-98.  Pradhan, et al (2002). JAMA 288:980-7.  Puerta-Vazquez R, et al (2004). Metabolism 53:59-65.  Ramirez-Tortosa, et al (1999). J Nutr 129:2177-83.  Ridker, et al (2000a). N Engl J Med 342:836-43.  Ridker et al (2000b). Circulation 101:1767–72.  Ros E (2003). Am J Clin Nutr 78(suppl):617S-25S.  Sabaté J (2003). Am J Clin Nutr 78(suppl):647S-50S.  Sanchez-Villegas A, et al (2003). Eur J Clin Nutr 57:285-92.  Singh RB, et al (2002). Lancet 360:1455-61.  Tanne, et al (2002). Stroke 33:2182-6.  Trichopoulou A, et al (1995). BMJ 311:1457-60.  Trichopoulou A, et al (2003). N Engl J Med 348:2599-608.  Tunstall-Pedoe H, et al (1999). Lancet 353:1547-57.  USDA (2000). Nutrition and Your Health: Dietary Guidelines for Americans. Washington: US Department of Agriculture. http://www.health.gov/ dietaryguidelines/ dga2000/ document/ contents.htm.  Visioli F, Galli C (1998). Nutr Rev 56:142-7.  Visioli F, et al (2000). Circulation 102:2169-71.  Visioli F, Galli C (2002). Crit Rev Food Sci Nutr 42:209-21.  Willett WC, et al (1995). Am J Clin Nutr 61(Suppl):1402-6S.  Willett WC (2000). Proc Soc Exp Biol Med 225:187-90.  World Health Organization (1993). Health for All: statistical database. Geneva: WHO, Regional Office for Europe.  World Health Organization (2003a). Neglected Global Epidemics: three growing threats. In: WHO: The world health report 2003 - shaping the future. Geneve: WHO.  World Health Organization (2003b). Joint WHO/FAO Expert Consultation on Diet, Nutrition and the Prevention of Chronic Diseases. Geneva. WHO technical report series; 916.  Yaqoob, et al (1998). Am J Clin Nutr 67:129-35. |
| --- | --- |

Appendix. Quantitative Score of Compliance with the Mediterranean Diet

|  | Foods and frequency of consumption | Criteria for 1 point* |
| --- | --- | --- |
| 1 | Do you use olive oil as main culinary fat? | Yes |
| 2 | How much olive oil do you consume in a given day (including oil used for frying, salads, out of house meals, etc.)? | 4 or more tablespoons |
| 3 | How many vegetable servings do you consume per day?  *(1 serving = 200g - consider side dishes as 1/2 serving)* | 2 or more *(at least 1 portion raw*  *or as salad)* |
| 4 | How many fruit units (including natural fruit juices) do you consume per day? | 3 or more |
| 5 | How many servings of red meat, hamburger, or meat products (ham, sausage, etc.) do you consume per day? *(1 serving = 100-150 g)* | Less than 1 |
| 6 | How many servings of butter, margarine, or cream do you consume per day? *(1 serving = 12 g)* | Less than 1 |
| 7 | How many sweet/carbonated beverages do you drink per day? | Less than 1 |
| 8 | How much wine do you drink per week? | 7 or more glasses |
| 9 | How many servings of legumes do you consume per week?  *(1 serving = 150 g)* | 3 or more |
| 10 | How many servings of fish or shellfish do you consume per week?  (1 serving: 100-150 g fish, or 4-5 units or 200 g shellfish) | 3 or more |
| 11 | How many times per week do you consume commercial sweets or pastries (not homemade), such as cakes, cookies, biscuits, or custard? | Less than 3 |
| 12 | How many servings of nuts (including peanuts) do you consume per week?  *(1 serving = 30 g)* | 1 or more |
| 13 | Do you preferentially consume chicken, turkey o rabbit meat instead of veal, pork, hamburger or sausage? | Yes |
| 14 | How many times per week do you consume vegetables, pasta, rice, or other dishes seasoned with *sofrito* (sauce made with tomato and onion, leek, or garlic, simmered with olive oil)? | 2 or more |

* 0 points if these criteria are not met.
